# Effectiveness of online mindfulness-based interventions in improving mental health during the COVID-19 pandemic: A systematic review and meta-analysis of randomized controlled trials

**DOI:** 10.1371/journal.pone.0274177

**Published:** 2022-09-21

**Authors:** Bendix Samarta Witarto, Visuddho Visuddho, Andro Pramana Witarto, Damba Bestari, Brihastami Sawitri, Tando Abner Sivile Melapi, Citrawati Dyah Kencono Wungu

**Affiliations:** 1 Medical Program, Faculty of Medicine, Universitas Airlangga, Surabaya, Indonesia; 2 Department of Psychiatry, Faculty of Medicine Universitas Airlangga/Dr. Soetomo Hospital, Surabaya, Indonesia; 3 Department of Psychiatry, Universitas Airlangga Hospital, Surabaya, Indonesia; 4 Department of Psychiatry, University of the Witwatersrand, Johannesburg, South Africa; 5 Department of Physiology and Medical Biochemistry, Faculty of Medicine, Universitas Airlangga, Surabaya, Indonesia; 6 Institute of Tropical Disease, Universitas Airlangga, Surabaya, Indonesia; Duta Wacana Christian University School of Medicine / Bethesda Hospital, INDONESIA

## Abstract

**Introduction:**

Psychotherapies, such as mindfulness-based interventions (MBIs), are currently needed to tackle mental health problems. Online MBIs have become promising since face-to-face interventions are limited during the COVID-19 pandemic due to lockdown and social distancing. This systematic review and meta-analysis aimed to investigate the effect of online MBIs in improving mental health, mainly depression, anxiety, and stress.

**Materials and methods:**

A systematic literature search was conducted according to the PRISMA 2020 guidelines on several databases for eligible studies up to October 17, 2021. Study quality was assessed using the Cochrane’s Risk of Bias 2 tool. Effect sizes were presented as standardized mean difference (Hedges’ *g*) between the online MBIs and control groups at post-test and follow-up using a random-effects model.

**Results:**

Eight randomized controlled trials involving 868 participants were included in this meta-analysis. The pooled adherence rate to online MBIs was 94% (95% CI = 91% to 98%). The findings revealed that online MBIs had a statistically significant small to moderate effect in reducing depression (*g* = -0.32; 95% CI = -0.49 to -0.14; *I*^2^ = 0%), a small effect on anxiety (*g* = -0.25; 95% CI = -0.43 to -0.06; *I*^2^ = 27%), and a moderate effect on stress (*g* = -0.62; 95% CI = -1.09 to -0.16; *I*^2^ = 83%). In addition, significant small effects at follow-up were observed for depression (*g* = -0.26; 95% CI = -0.48 to -0.04; *I*^2^ = 0%) and anxiety (*g* = -0.28; 95% CI = -0.48 to -0.08; *I*^2^ = 0%), but not for stress.

**Conclusion:**

Online MBIs have beneficial effects on mental health, particularly depression, anxiety, and stress, during the COVID-19 pandemic. Given the limitations of the current study, future trials that specifically consider potential effect influencing factors, longer follow-up evaluation, and methodological quality are warranted.

## Introduction

The Coronavirus Disease 2019 (COVID-19) pandemic has globally affected the community’s mental health and heightened its prevalence, particularly for depression, anxiety, and stress [1–3]. This influence on mental health may be caused by the social restrictions, lockdowns, limitations in economic and educational activities, loss of livelihood, and shifting priorities of governments in order to control COVID-19 outbreaks. Additionally, social life has shifted into the online world as a part of adaptation during the pandemic [4]. Social media and online collaboration tools have become crucial for maintaining interaction and activities [5]. Unfortunately, the problematic use of these digital tools may further cause a negative impact on mental health and emotional well-being [6]. A recent worldwide meta-analysis showed that 27.6%, 32.6%, and 30.3% of the population suffered from depression, anxiety, and stress-related insomnia, respectively, during the COVID-19 pandemic [7]. The prevalence of depression and anxiety was higher in Asia than in Europe (35.3% vs. 32.4% and 32.9% vs. 23.8%, respectively), while the prevalence of stress in Europe was higher than in Asia (31.9% vs. 27.9%) [8]. Concerns regarding these mental health issues have grown since the start of the crisis. Hence, mental health interventions are urgently needed to tackle these problems [2].

A previous review has demonstrated evidence on the effectiveness of mindfulness-based intervention (MBI) programs in reducing some psychological symptoms, including depression, anxiety, stress, insomnia, addiction, psychosis, and post-traumatic stress disorder [9]. The MBI is known as one of several psychological intervention methods specifically designed for mental health. It works by improving the participants’ ability to observe their inner thoughts or feelings oriented to their acceptance of life experiences [10]. Furthermore, MBI was also beneficial to teach the participants to address their thoughts and emotions in a non-judgmental way [11]. Mindfulness practice was first incorporated into Western medicine for chronic pain treatment in 1982 by Jon Kabat-Zinn and was derived from Buddhist meditation traditions [12]. Since then, MBI practices in mental health management, specifically in alleviating one’s emotional states, have been introduced in several modified methods, i.e., Mindfulness-Based Stress Reduction (MBSR), Mindfulness-Based Cognitive Therapy (MBCT), Dialectical Behaviour Therapy (DBT), and Acceptance and Commitment Therapy (ACT) [10]. Of those, the two most commonly adopted MBI programs are the MBSR and MBCT, which consist of eight weekly mindfulness sessions and a one-day retreat [9]. Both MBSR and MBCT have demonstrated an effective reduction in the three most common mental health problems, which are depression, stress, anxiety, and some physical health outcomes related to chronic diseases [10, 13].

During the COVID-19 pandemic, the face-to-face intervention method becomes limited due to quarantine, lockdown, and social distancing [13]. Many online platforms have been used to deliver health interventions, including programs in the field of mental health [10, 13]. Several advantages of online mental health interventions as compared to offline or traditional face-to-face methods have been addressed: (1) easily accessible; (2) more suitable for people in any environment without significantly interfering with their work pace; (3) some may not necessarily require the direct involvement of a knowledgeable therapist; and (4) participants may access the treatment program information at any time outside the schedule and further facilitate more retention [14]. Moreover, online MBIs may be considered even more cost-effective than face-to-face MBIs [9, 14]. Many smartphone applications additionally offer some mindfulness-based practices [12]. Thus, these features allow further access to online MBI programs. Online delivery methods in mental health interventions are also helpful for clinicians and psychologists to reach better mental health care for the general population, since nowadays people tend to be more comfortable with internet-based interventions [15]. Various studies have been published on the effectiveness of online MBIs; however, no meta-analyses have evaluated it, particularly in the setting of the COVID-19 pandemic. Therefore, in this exploratory meta-analysis, we aim to estimate the effect of online MBIs in improving mental health outcomes, mainly depression, anxiety, and stress. This review focuses on the two most used MBI methods (i.e., MBCT and MBSR) in randomized clinical trials (RCTs) conducted amid the COVID-19 pandemic.

## Materials and methods

This systematic review and meta-analysis were conducted in conformity with the Preferred Reporting Items for Systematic Reviews and Meta-Analyses (PRISMA) 2020 guidelines [16]. The detailed protocol of the current study has been previously registered and published on the International Prospective Register of Systematic Reviews (PROSPERO) (https://www.crd.york.ac.uk/prospero/) with the registration number CRD42021287616 [17].

### Search strategy

A computerized systematic literature data searching of relevant studies was conducted in PubMed, Scopus, Web of Science, ProQuest, Cumulative Index to Nursing and Allied Health Literature (CINAHL) via EBSCO, the Cochrane Central Register of Controlled Trials (CENTRAL), and the World Health Organization (WHO) COVID-19 Research Databases for studies published up to October 17, 2021. We additionally searched for grey literatures, specifically preprints, using MedRxiv and BioRxiv as the source databases. In systematic reviews and meta-analyses, grey literatures may help reduce the impact of publication bias, increase the comprehensiveness of the search, and provide a balanced view of the available evidence and more accurate effect sizes [18, 19]. It is also recognized that research sharing on the study of COVID-19 using preprints has increased rapidly during the pandemic [20]. At the same time, we performed a manual hand-search on Google Scholar and the reference lists of the included studies to maximize the search results. The following main keywords were initially established: "mindfulness", "online", "COVID-19", and "randomized controlled trial". We subsequently added several Medical Subject Headings (MeSH), thesaurus, and other free-text terms to construct database-specific search terms. The full search strings for each database are provided in **S1 Table in S1 File**. No language and publication date restrictions were set in all searches.

### Selection of studies

All search results were pooled and managed collectively using Google Sheets (https://docs.google.com/spreadsheets/) (Google LLC, Mountain View, CA, USA). After removing the duplicates, the articles were filtered by reviewing their title. Potentially relevant articles were then screened based on the abstract. All articles included in the next screening step were sought for retrieval. Afterwards, studies with available and published full-texts were retrieved and thoroughly assessed according to the pre-specified eligibility criteria. The reasonings of exclusion for all articles on each screening step were declared as appropriate in the spreadsheet. The initial literature searches and overall study selection process were performed independently by two investigators (BS and VV). Any disagreements were resolved in a consensus involving a third independent investigator (AP).

### Eligibility criteria

We adopted the Population, Intervention, Comparison, Outcome, and Study Design (PICOS) framework [21] (**S2 Table in S1 File**) as a guide in formulating and establishing the eligibility criteria of the current study. To be included in the systematic review and meta-analysis, studies had to meet the following criteria: (1) the study population consisted of subjects aged 18 years or older; (2) employed MBIs (MBSR or MBCT); (3) delivered the intervention entirely using online method via internet-based platforms or applications; (4) used either an active (received other type of intervention) or inactive (no intervention) control group; (5) used validated instruments to assess mental health outcome measures of depression, anxiety, and stress; (6) used a randomized controlled design; and (7) the study course (from recruitment phase to the end of intervention) was fully conducted during the period of the COVID-19 pandemic (the start of the COVID-19 pandemic was defined according to the WHO announcement on March 11, 2020 [22]). We accepted studies published in any language.

Studies were excluded if: (1) the full-text was irretrievable; (2) the intervention also involved other forms of psychotherapy unrelated to mindfulness practice, making it difficult to judge the absolute effect of MBI; or (3) the study did not report mean and standard deviation values to calculate the meta-analytic effect size and the authors did not respond after they were contacted for data request or were not willing to provide the data.

### Data extraction and quality assessment

Two investigators (BS and VV) independently performed data extraction from each study, using a pre-specified form tabulated within the spreadsheet. These were checked by the third investigator (AP). Disagreements were resolved through a discussion. The following relevant data were extracted: the first author, year of publication, study location (country and region), type of study population, sample size per study arm, gender (% of females), age, adherence rate to MBI, type of MBI (MBSR or MBCT), type of control group (active or inactive), delivery method (e.g., videoconferencing, website, or mobile application), guidance (with or without), number of sessions, length of intervention, time of measurements (post-intervention or follow-up), type of analysis (intention to treat [ITT] or per-protocol [PP]), and type of instruments used to assess outcome measures (depression, anxiety, or stress) along with the mean and standard deviation values per time of measurements. We defined adherence rate as the percentage of participants that completed all online MBI sessions compared to the baseline allocated participants in the intervention group. Studies that were not reported in the English language were translated using available online translators prior to data extraction. In case specific data were unavailable for extraction or not clearly reported in the paper, we contacted the author of the corresponding study. Finally, three authors were contacted, two of whom successfully responded and provided the data on request.

Two reviewers (BS and VV) then independently conducted a methodological quality assessment to evaluate the risk of bias of each eligible study using the Cochrane Collaboration’s Risk of Bias 2 (RoB 2) tool [23]. Discordance of judgments were resolved simultaneously through a group discussion involving a third reviewer (AP). The RoB 2 is a revised tool consisting of five bias domains explicitly designed to consider the risk of bias of randomized trials arising from: (1) the randomization process; (2) deviations from intended interventions; (3) missing outcome data; (4) the measurement of the outcome; and (5) the selection of the reported result. The risk of bias on each domain was rated as low risk, high risk, or some concerns (unclear) according to the algorithms that incorporated several domain-specific signalling questions. The levels of judgments from all domains were later deduced into an overall risk of bias for each study. Studies were judged with a low overall risk of bias if all domains presented with low risk. If at least one domain was rated as unclear, studies were judged as having some concerns. Studies were judged to be at high risk of bias if at least one domain showed a high risk or there were some concerns in multiple domains that could considerably lower the confidence of the study’s result.

### Statistical analysis

#### Main analysis

All analyses were conducted using Review Manager version 5.4 (The Cochrane Collaboration, The Nordic Cochrane Centre, Copenhagen, Denmark) and STATA version 16.0 (Stata Corporation, College Station, TX, USA). We performed separate quantitative analyses to determine the effect of MBIs on three different mental health outcomes: depression, anxiety, and stress. In the case where a study used more than one instrument to measure depression, anxiety, and stress, we used the most valid instrument so that each study outcome had a single effect size. Comparison of means and standard deviations (SDs) between intervention and control groups measurements at post-test were analyzed as the primary outcomes of this study. We additionally performed secondary analyses for outcomes comparing between-groups means and SDs of depression, anxiety, and stress at follow-up measurements. The ITT analyses were preferred over PP if studies reported both types of analysis. Furthermore, to understand the overall adherence rate of participants to online MBIs, we conducted a meta-analysis of proportions using the double arcsine transformation method [24]. For all outcomes, we performed meta-analyses with and without outliers. A study was considered an outlier if its 95% confidence interval (CI) was located outside of the 95% CI of the pooled effect by visually examining it on the forest plot [25]. Forest plots were constructed only for the meta-analyses with outliers.

In this study, we did not perform analyses using change-from-baseline (pre-to-post and pre-to-follow-up differences) between-groups scores since this practice is not recommended by Cochrane [26]. Although this method could remove the component of between-subject variability from the analysis, it involves an additional calculation, in which it is considered more inefficient. Furthermore, due to the nature of randomized trials, as the type of studies included in this meta-analysis, we could assume in theory that comparison of final measurement (at post-test and follow-up) has approximately the same effect as the comparison of changes from baseline [27].

We applied the standardized mean difference (SMD) as the summary statistics of the primary and secondary outcomes since the included studies used different psychometric instruments to assess depression, anxiety, and stress. The SMD represents the difference in means between the intervention and control groups divided by the combined SD. We used Hedges’ *g* as the standard metric of the effect size in all analyses. The Hedges’ *g* is a type of effect size for SMD, which includes an additional adjustment for small sample studies. The pooled effect of intervention using Hedges’ *g* is interpreted as similar to Cohen’s *d*, where 0.2 represents small, 0.5 represents moderate, and 0.8 represents large effect sizes [28].

Heterogeneity between studies was examined with the Cochran’s *Q* statistic and quantified using the Higgins’ *I*^2^ statistic, where *I*^2^ values of 0%, 25%, 50%, and 75% indicated negligible, low, moderate, and high heterogeneity, respectively [29]. Given that there was a considerable variability and diversity between studies interventions and characteristics of the study population, we primarily applied the random-effects model for all analyses. A *p*-value of < 0.05 was used to indicate statistical significance in all analyses.

#### Analysis of publication bias

Publication bias was assessed visually using the inverted funnel plot and quantitatively using the Egger’s regression test [30] to detect small study effects and other potential reporting biases. Small-study effects are described as the tendency of smaller studies to result in a more significant intervention effect than the large ones. Consequently, these studies would be more likely to be published. In addition, publication bias could exist due to the tendency of authors and journals to publish studies with significant results [31]. These phenomena will result in the asymmetrical distribution in the funnel plot analysis and a significant Egger’s test result.

#### Sensitivity, subgroup, and meta-regression analyses

We prioritized performing sensitivity, subgroup, and meta-regression analyses only for meta-analyses that evaluated the primary outcomes (including outliers). Sensitivity analyses were carried out by excluding each study from the analysis one at a time (leave-one-out method). Subgroup and meta-regression analyses were performed to evaluate the differences in effect sizes between subgroups and the effect of certain covariates on the pooled outcome. We conducted subgroup analyses based on: (1) study location (Asia vs. non-Asia); (2) characteristics of the study population (general vs. non-general population); (3) type of MBI (MBSR vs. MBCT); (4) type of control group (active vs. inactive); (5) delivery method (videoconferencing vs. non-videoconferencing); and (6) guidance (with vs. without). Subgroup analyses were performed only if each subgroup in the analyses contained at least two studies. Restricted maximum likelihood random-effects meta-regression were carried out for: (1) mean of age; (2) % of females; (3) number of sessions; and (4) length of intervention in weeks. Meta-regression analyses that presented significant results were visualized through bubble plots.

## Results

### Overview of study selection

**Fig 1:**
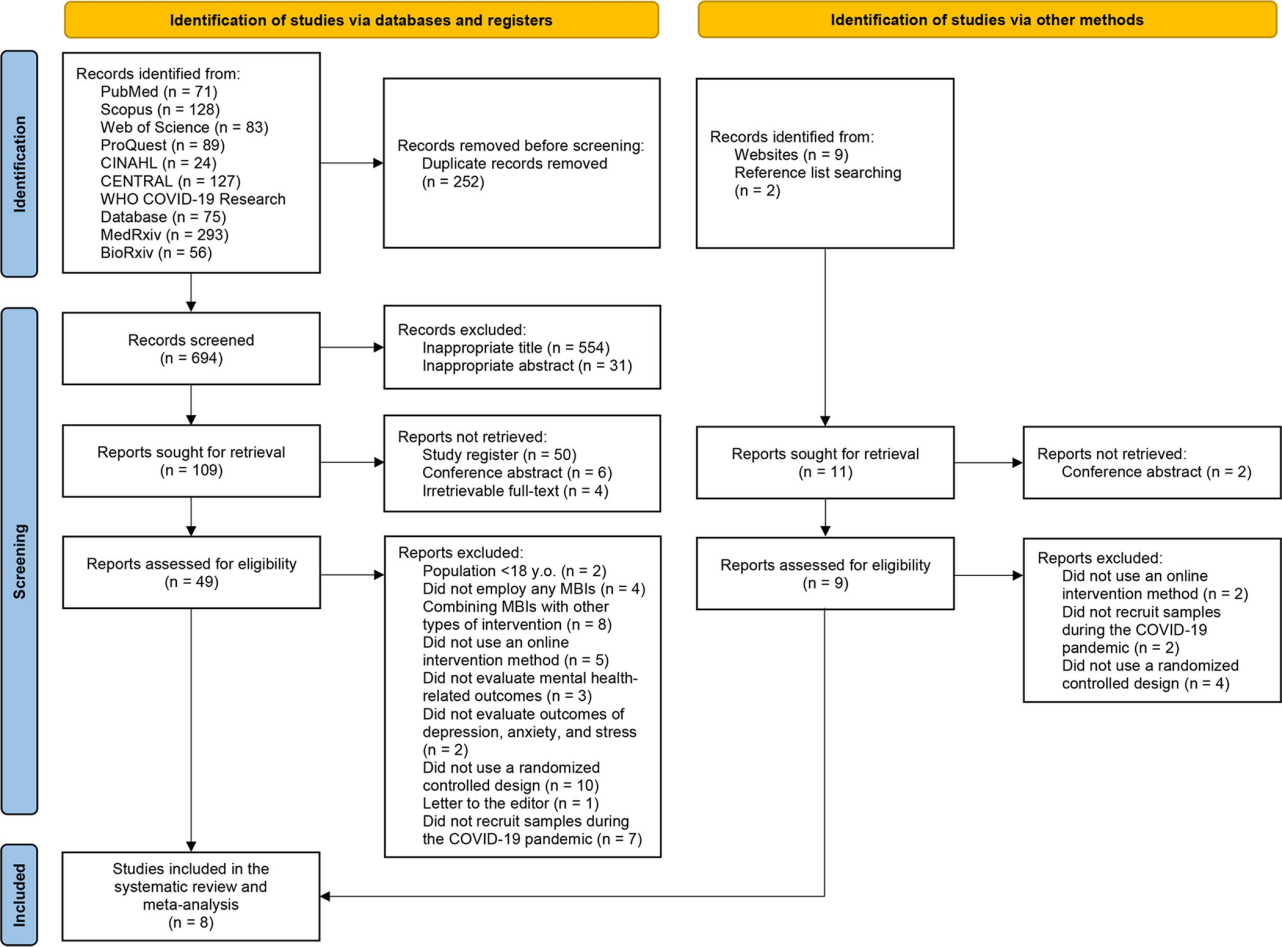
PRISMA flow diagram of the study selection process. **CENTRAL**, Cochrane Central Register of Controlled Trials; **CINAHL**, Cumulative Index to Nursing and Allied Health Literature; **COVID-19**, Coronavirus Disease 2019; **WHO**, World Health Organization.

A PRISMA flow diagram of the overall study selection process is illustrated in **Fig 1**. The initial electronic database searches yielded 946 hits. A total of 252 duplicates were then removed. After reviewing 694 records, 554 and 31 were excluded based on the titles and abstracts, respectively. A total of 60 reports identified as study registers, conference abstracts, or with no access to full-texts were not retrieved. Afterwards, we thoroughly assessed the remaining 49 full-texts for eligibility and 42 were further excluded due to the irrelevancy of their population characteristics, type and method of interventions, reported and evaluated outcomes, study design, or samples recruitment period. In addition to database searching, we identified 11 extra records from websites and reference lists searching. Of those, two reports were not retrieved. After judging the eligibility of the remaining reports, eight were excluded. Accordingly, the overall screening process led to the inclusion of 8 RCTs [32–39] in this systematic review and meta-analysis.

### Characteristics and outcomes of included studies

The characteristics of the included studies are summarized in **Table 1**. The 8 RCTs yielded a total of 868 adult participants (mean age: 22 to 36 years), from which 451 received the MBIs, and 417 were control samples. The study sample sizes were variable ranging from 51 to 183. All studies were conducted during the COVID-19 pandemic. Females accounted for more than half of the study population in all studies. Half of the studies (*n* = 4) were located in Asia, and the rest were in America (*n* = 3) and Europe (*n* = 1). The studies included diverse type of population: general (unspecified) population (*n* = 2), university students (*n* = 3), healthcare workers (*n* = 1), social workers (*n* = 1), and obstetrics and gynecology outpatients (*n* = 1). Two studies examined MBSR, five MBCT, and one employed a combination of MBSR and MBCT intervention. In three studies, the duration of MBSR and MBCT interventions were conducted according to the original protocol in eight weekly sessions, while the other studies used a modified protocol. The MBIs were varied from 1 to 10 sessions, with the length of intervention ranging from 1 to 8 weeks. The interventions were most commonly delivered through a videoconference (*n* = 4). Other methods included a website (*n* = 1) and a mobile application (*n* = 3). The interventions in four studies were guided by either a specialist in MBIs or a trained therapist. An inactive control condition was used in five RCTs, all of which were waitlist groups. The three remaining RCTs used an active control, in which participants received access to mental health education (*n* = 1), social support-based condition (*n* = 1), or were asked to answer manipulated questions (*n* = 1).

**Table 1:** Characteristics of included studies.

First Author, Year	Study Location	Characteristics of Population	Baseline Sample Size		% Female^a^	Age^b^	Type of MBI	Type of Control Group	Delivery Method	Guidance	Number of Sessions	Length of Intervention	Measurements	Type of Analysis	Instrument of Outcome Measures		
			Online MBIs	Control											Depression	Anxiety	Stress
**Alvarado-García et al., 2021 [32]**	Peru, America	General population	30	30	N/A	N/A	MBSR	Inactive (Waitlist)	Videoconferencing	With	8 sessions	8 weeks	Post	ITT	N/A	N/A	PSS-10
**Hosseinzadeh Asl, 2021 [33]**	Turkey, Asia	Social workers	30	21	55.1	33.06 ± 6.02	MBCT	Inactive (Waitlist)	Videoconferencing	With	4 sessions	4 weeks	Post, 1-month follow-up	PP	DASS-21-D	DASS-21-A	DASS-21-S
**Huang et al., 2021 [34]**	China, Asia	Healthcare workers in COVID-19 isolation wards	58	60	77.1	32.27 ± 6.42	MBSR	Active (Mental health education)	Videoconferencing	With	8 sessions	8 weeks	Post	ITT	N/A	N/A	SRQ
**Kam et al., 2021 [35]**	Canada, America	University students	34	30	83.9	29.9 ± 8.8	MBCT	Inactive (Waitlist)	Mobile application	Without	10 sessions	2 weeks	Post	PP	PROMIS-D	PROMIS-A	N/A
**Pheh et al, 2020 [36]**	Malaysia, Asia	General population	104	79	73.9	28.75 ± 9.01	MBCT	Active (Answering manipulated questions)	Website	Without	1 session	1 week	Post, 2-week follow-up	PP	N/A	GAD-7	SUDS
**Simonsson et al., 2021 [37]**	England, Europe	University students	88	89	64.4	23.27 ± 5.60	MBCT	Inactive (Waitlist)	Videoconferencing	With	8 sessions	8 weeks	Post, 1-month follow-up	PP	PROMIS-D	PROMIS-A	N/A
**Smith et al., 2021 [38]**	United States, America	Obstetrics and gynecology outpatients	50	51	100	36.21 ± 11.30	MBCT	Inactive (Waitlist)	Mobile application	Without	4 sessions	4 weeks	Post	ITT	HADS-D	HADS-A	PSS-10
**Sun et al., 2021 [39]**	China, Asia	University students	57	57	73.7	22.21 ± 2.67	MBSR and MBCT	Active (Social support)	Mobile application	With	4 sessions	4 weeks	Post, 2-month follow-up	ITT	PHQ-9	GAD-7	N/A

^a^% female of the total study population at baseline.

^b^Mean and standard deviation of the study population age at baseline.

**COVID-19**, Coronavirus Disease 2019; **DASS-21-A**, Depression, Anxiety, and Stress Scale-21 Anxiety Subscale; **DASS-21-D**, Depression, Anxiety, and Stress Scale-21-Depression Subscale; **DASS-21-S**, Depression, Anxiety, and Stress Scale-21-Stress Subscale; **GAD-7**, Generalized Anxiety Disorder-7; **HADS-A**, Hospital Anxiety and Depression Scale-Anxiety Subscale; **HADS-D**, Hospital Anxiety and Depression Scale-Depression Subscale; **ITT**, intention-to-treat; **MBCT**, Mindfulness-Based Cognitive Therapy; **MBI**, mindfulness-based intervention; **MBSR**, Mindfulness-Based Stress Reduction; **N/A**, not available; **PHQ-9**, Patient Health Questionnaire-9; **PP**, per-protocol; **PROMIS-A**, Patient-Reported Outcomes Measurement Information System-Anxiety Subscale; **PROMIS-D**, Patient-Reported Outcomes Measurement Information System-Depression Subscale; **PSS-10**, Perceived Stress Scale-10; **SD**, standard deviation; **SRQ**, Stress Response Questionnaire; **SUDS**, Subjective Unit of Distress Scale.

Among the 8 RCTs, half reported outcomes using ITT analyses. The follow-up times after intervention in four studies were varied, ranging from 2 weeks to 2 months. All studies used validated instruments to measure depression, anxiety, and stress. Outcome measures of depression were available in 5 studies, anxiety in 6, and stress in 5 studies. **S3 Table in S1 File** provides the outcome values reported in each included study.

### Study quality assessment

**Fig 2:**
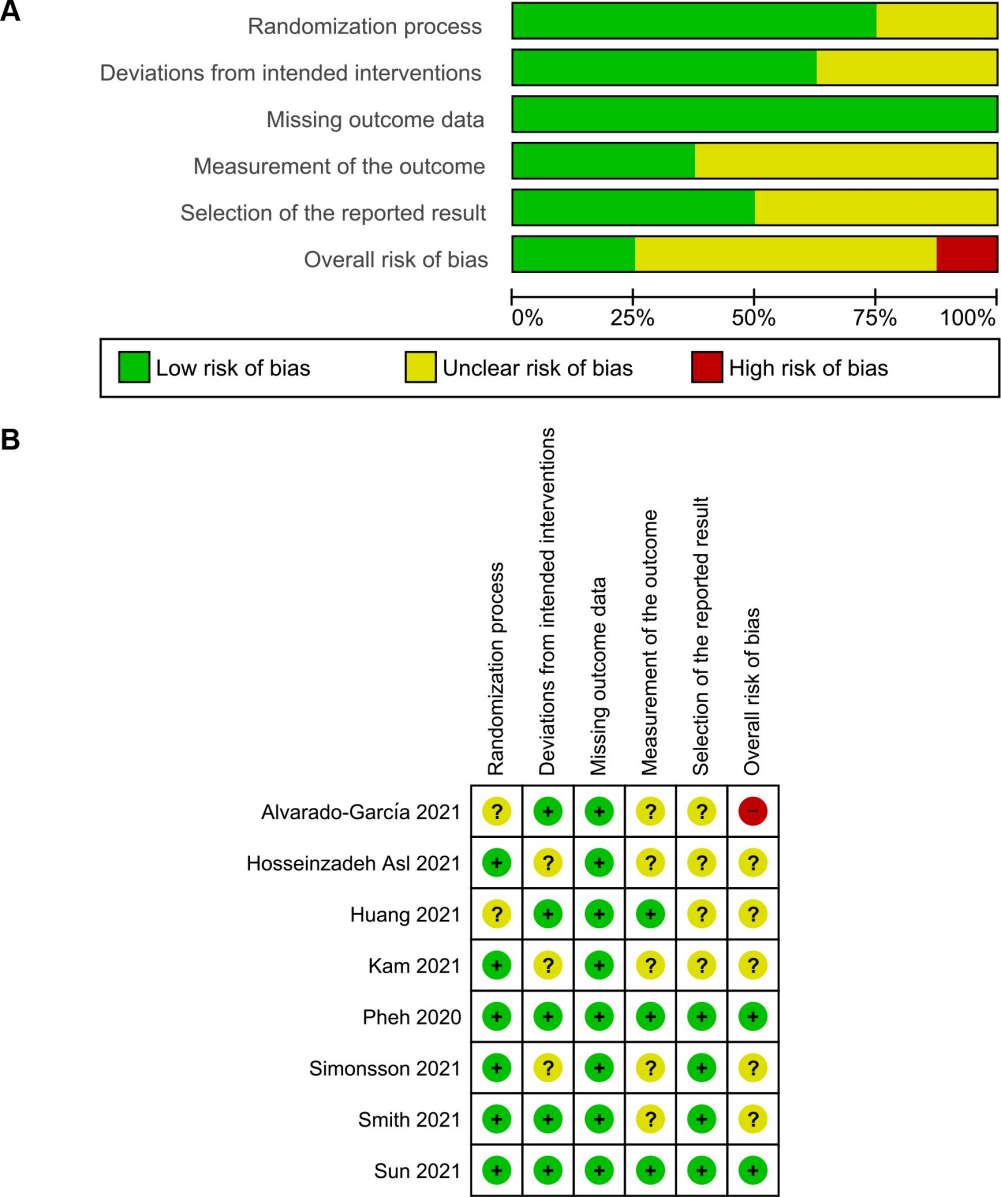
Results of quality assessment of included studies using the Risk of Bias 2 tool. (**A**) Domain-specific quality assessment graph. (**B**) Detailed quality assessment summary.

The result of the domain-specific quality assessment is provided in **Fig 2A**, while the detailed risk of bias evaluation of each study is summarized in **Fig 2B**. According to the Cochrane’s RoB 2 tool, two studies were rated as low risk in all domains, thus having a low overall risk of bias. Five RCTs were rated as having some concerns, from which one study had unclear risk in 1 domain, while two studies in 2, and two studies in 3 domains. One RCT, i.e., Alvarado-García et al. [32], was rated as high risk since it was judged to have some concerns for multiple domains that might lower confidence of the study’s result. If viewed from each domain individually, two studies reported incomplete information regarding the randomization process, and thus were rated as unclear risk of bias. There were some concerns of deviations from the intended interventions in three studies due to the lack of information on blinding procedures. All RCTs were rated as low of risk in the domain of bias due to missing outcome data. Some concerns were observed during the measurement of outcome in five studies since there was a potential influence by the knowledge of intervention status. Four studies had an unclear risk of bias in the selective reporting domain due to the unavailability of the pre-published trial protocol, and hence we could not judge the reliability of the reported results.

### Adherence rate

**Fig 3:**
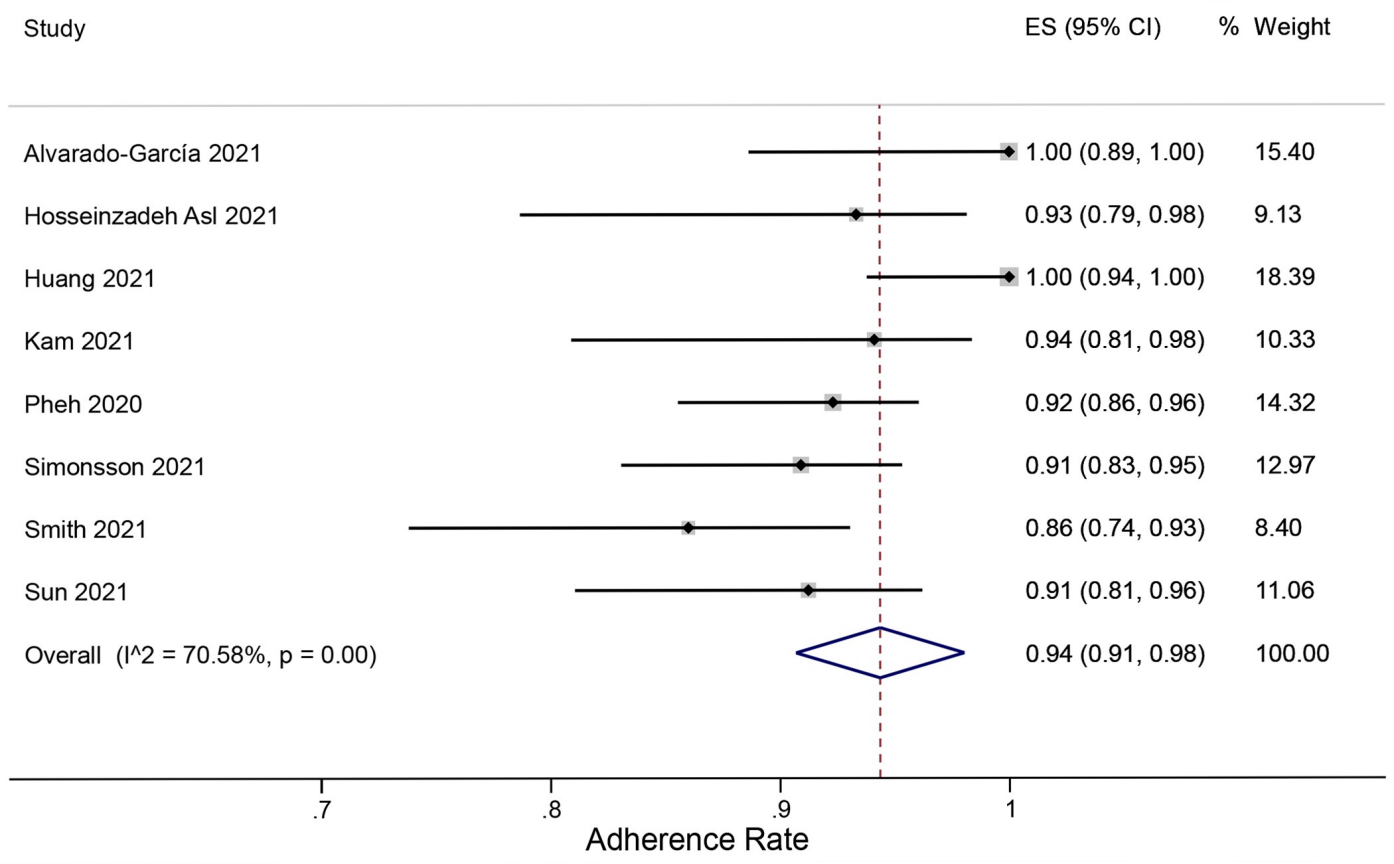
Forest plot of meta-analysis of proportions for adherence rate to online MBIs.

When adherence was defined as the completion of all sessions, the rates from 8 RCTs were varied, ranging from 86% to 100%. The overall adherence rate to online MBIs obtained from meta-analysis was 94% (95% CI = 91% to 98%) with high heterogeneity (*I*^2^ = 70.6%). No study outliers were detected. The forest plot of pooled adherence rate analysis is presented in **Fig 3**.

### Primary outcomes (effects at post-intervention)

#### Effects on depression

**Fig 4:**
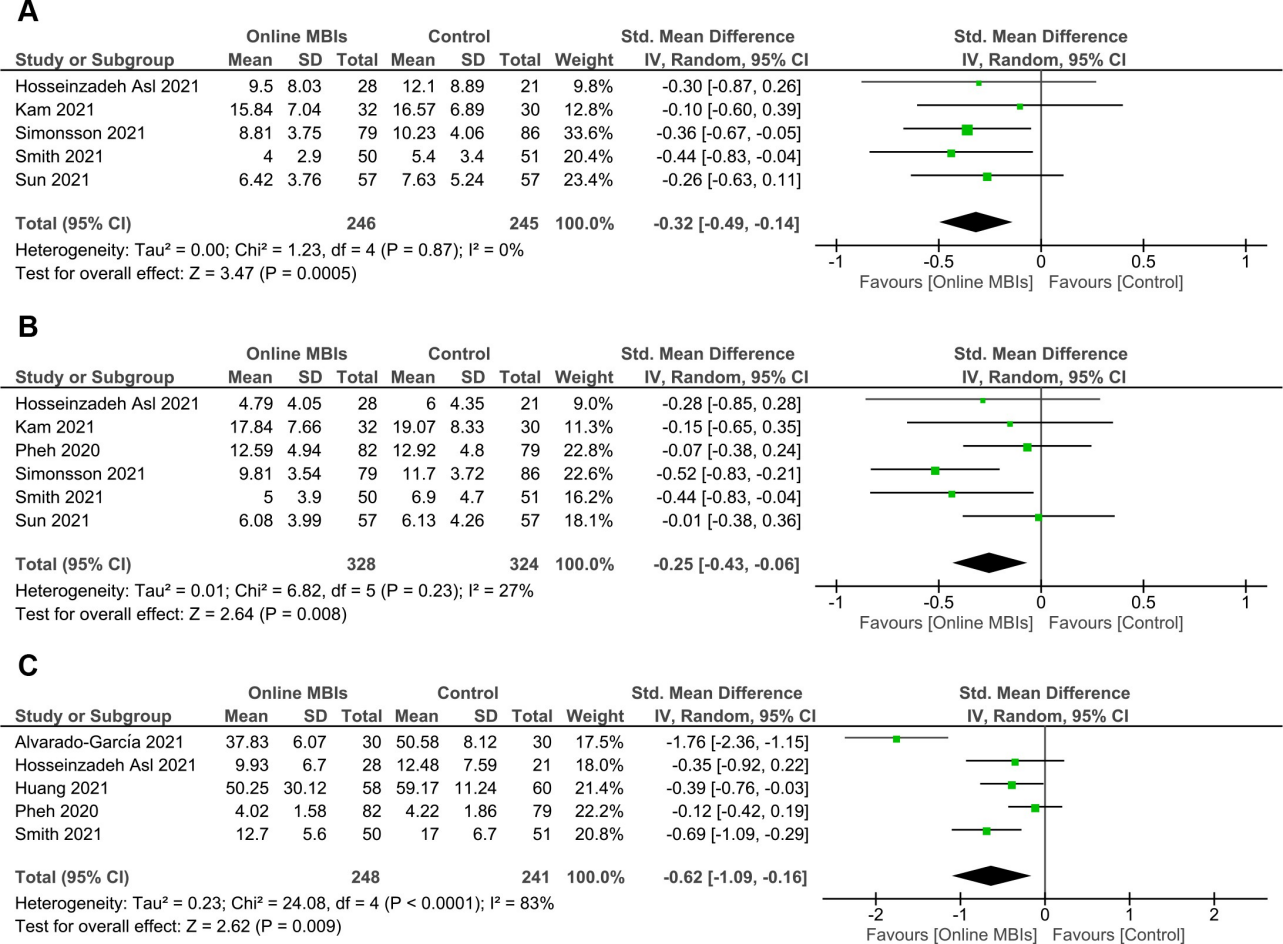
Forest plots of meta-analyses of online MBIs effect on mental health outcomes at post-intervention. (**A**) Depression. (**B**) Anxiety. (**C**) Stress.

A total of 246 online MBIs and 245 control participants from 5 studies were included in the meta-analysis for depression outcome (**Fig 4A**). Online MBIs during the COVID-19 pandemic showed a significant, small to moderate effect in reducing depression at post-intervention (*g* = -0.32; 95% CI = -0.49 to -0.14; *p* < 0.001). The level of heterogeneity was negligible (*I*^2^ = 0%). No outliers were detected based on the forest plot. Sensitivity analysis using the leave-one-out method revealed that no study had a substantial influence on the significance of the overall effect size.

#### Effects on anxiety

Six RCTs with a total of 328 participants receiving online MBIs and 324 controls were analyzed to determine the pooled effect on anxiety (**Fig 4B**). A significant, small reduction in anxiety was observed at post-intervention (*g* = -0.25; 95% CI = -0.43 to -0.06; *p* = 0.008). The level of heterogeneity was low (*I*^2^ = 27%). Examination of the forest plot showed no potential study outliers. After removing one study by Simonsson et al. [37] in sensitivity analysis, the pooled effect was reduced to insignificance.

#### Effects on stress

For stress outcome, we successfully included five studies involving 248 online MBIs and 241 control subjects (**Fig 4C**). The overall effect on stress after online MBIs was significant with *g* = -0.62 (95% CI = -1.09 to -0.16; *p* = 0.009). This effect was considered moderate. A high level of heterogeneity was observed (*I*^2^ = 83%). After examining the forest plot, we identified one potential outlier (Alvarado-García et al. [32]). Removal of the outlier resulted in a reduction of the pooled effect to *g* = -0.37 (95% CI = -0.62 to -0.11), but this effect remained significant (*p* = 0.005). Additionally, the heterogeneity shifted to a moderate level (*I*^2^ = 40%). A subsequent leave-one-out sensitivity analysis suggested that the significance of the overall effect was robust and not affected by any single study.

#### Subgroup analysis

Subgroup analyses for all post-intervention outcomes (including outliers) are presented in **Table 2**. For anxiety outcome, we found significantly larger effect sizes for studies conducted outside of Asia than in Asia (*p* = 0.03) and studies that used an inactive control than an active control condition (*p* = 0.02). No significant differences between subgroups were found for depression and stress. Subgroup analyses for depression, anxiety, and stress outcomes based on several covariates were not conducted due to the lack of studies included in each subgroup.

**Table 2:** Summary of subgroup analyses of online MBIs effect on mental health outcomes at post-intervention (including outliers).

Outcome Measure	Covariate	Subgroup	Number of Studies	Effect Size				Heterogeneity		*p*-value of Difference between Subgroups
				Hedges’ *g*	95% CI	*Z*	*p*-value	*I* ^2^	*p*-value	
**Depression**	Study region	Asia	2	-0.28	-0.59, 0.03	1.75	0.08	0%	0.91	0.76
		Non-Asia	3	-0.34	-0.55, -0.12	3.01	0.003	0%	0.57	
	Delivery method	Videoconferencing	2	-0.35	-0.62, -0.08	2.52	0.01	0%	0.86	0.75
		Non-videoconferencing	3	-0.29	-0.53, -0.05	2.40	0.02	0%	0.57	
	Guidance	With	3	-0.32	-0.54, 0.10	2.86	0.004	0%	0.92	0.95
		Without	2	-0.31	-0.63, 0.01	1.87	0.06	7%	0.30	
**Anxiety**	Study region	Asia	3	-0.08	-0.30, 0.14	0.72	0.47	0%	0.73	0.03^a^
		Non-Asia	3	-0.42	-0.64, -0.20	3.77	< 0.001	0%	0.47	
	Control type	Active	2	-0.04	-0.28, 0.19	0.37	0.71	0%	0.82	0.02^a^
		Inactive	4	-0.40	-0.61, -0.20	3.87	< 0.001	0%	0.64	
	Delivery method	Videoconferencing	2	-0.46	-0.74, -0.19	3.34	< 0.001	0%	0.48	0.06
		Non-videoconferencing	4	-0.15	-0.34, 0.04	1.55	0.12	0%	0.42	
	Guidance	With	3	-0.28	-0.62, 0.05	1.64	0.10	53%	0.12	0.69
		Without	3	-0.20	-0.43, 0.03	1.72	0.09	5%	0.35	
**Stress**	Study region	Asia	3	-0.25	-0.47, -0.03	2.24	0.02	0%	0.49	0.08
		Non-Asia	2	-1.20	-2.24, -0.16	2.25	0.02	88%	0.00	
	Population type	General population	2	-0.91	-2.52, 0.69	1.12	0.26	96%	< 0.001	0.61
		Non-general population	3	-0.50	-0.74, -0.25	3.98	< 0.001	0%	0.48	
	MBI type	MBSR	2	-1.05	-2.39, 0.28	1.54	0.12	93%	< 0.001	0.34
		MBCT	3	-0.37	-0.75, 0.00	1.94	0.05	60%	0.08	
	Control type	Active	2	-0.24	-0.51, 0.03	1.72	0.09	22%	0.26	0.09
		Inactive	3	-0.92	-1.66, -0.18	2.43	0.02	84%	0.002	
	Delivery method	Videoconferencing	3	-0.81	-1.64, 0.01	1.93	0.05	87%	< 0.001	0.40
		Non-videoconferencing	2	-0.39	-0.95, 0.17	1.35	0.18	80%	0.03	
	Guidance	With	3	-0.81	-1.64, 0.01	1.93	0.05	87%	< 0.001	0.40
		Without	2	-0.39	-0.95, 0.17	1.35	0.18	80%	0.03	

^a^ *p* < 0.05

**CI**, confidence interval; **MBCT**, Mindfulness-Based Cognitive Therapy; **MBI**, mindfulness-based intervention; **MBSR**, Mindfulness-Based Stress Reduction.

#### Meta-regression analysis

Meta-regression analysis showed that length of intervention (**S1A Fig in S1 File**) had a significant positive moderating effect on anxiety (*Z* = -2.09; *p* = 0.04), meaning that online MBIs with longer intervention would result in greater anxiety reduction. Furthermore, a significant positive influence of mean age on stress outcome was observed (*Z* = -2.23; *p* = 0.03), by which we found that online MBIs were more effective in reducing stress in older populations (**S1B Fig in S1 File**). All other covariates did not have a significant influence on each outcome. The results of meta-regression analyses (including outliers) are summarized in **Table 3**.

**Table 3:** Summary of meta-regression analyses of online MBIs effect on mental health outcomes at post-intervention (including outliers).

Outcome Measure	Covariate	Number of Studies	*Z*	*p*-value
**Depression**	Mean age	5	-0.29	0.77
	% female	5	-0.20	0.84
	Number of sessions	5	0.49	0.63
	Length of intervention	5	-0.59	0.55
**Anxiety**	Mean age	6	-0.37	0.71
	% female	6	-0.06	0.95
	Number of sessions	6	-1.21	0.23
	Length of intervention	6	-2.09	0.04^a^
**Stress**	Mean age	4	-2.23	0.03^a^
	% female	4	-1.36	0.17
	Number of sessions	5	-1.38	0.17
	Length of intervention	5	-1.38	0.17

^a^ *p* < 0.05

#### Publication bias

For depression (**S2A Fig in S1 File**) and anxiety (**S2B Fig in S1 File**), funnel plots showed a rather symmetrical distribution of studies, indicating no potential of publication bias. These findings were confirmed by insignificant Egger’s test results for depression (*Z* = 0.53; *p* = 0.59) and anxiety (*Z* = 0.10; *p* = 0.92). However, a skewed distribution towards a higher effect size was observed in the funnel plot for stress (**S2C Fig in S1 File**). The result of Egger’s test for stress also revealed the potential of publication bias (*Z* = -2.06; *p* = 0.04). Surprisingly, the removal of outlier resulted in a more symmetrical distribution of the funnel plot (**S2D Fig in S1 File**) and an insignificant Egger’s test (*Z* = -0.57; *p* = 0.57), suggesting that small study effects might arise from one study (Alvarado-García et al. [32]).

### Secondary outcomes (effects at follow-up)

**Fig 5:**
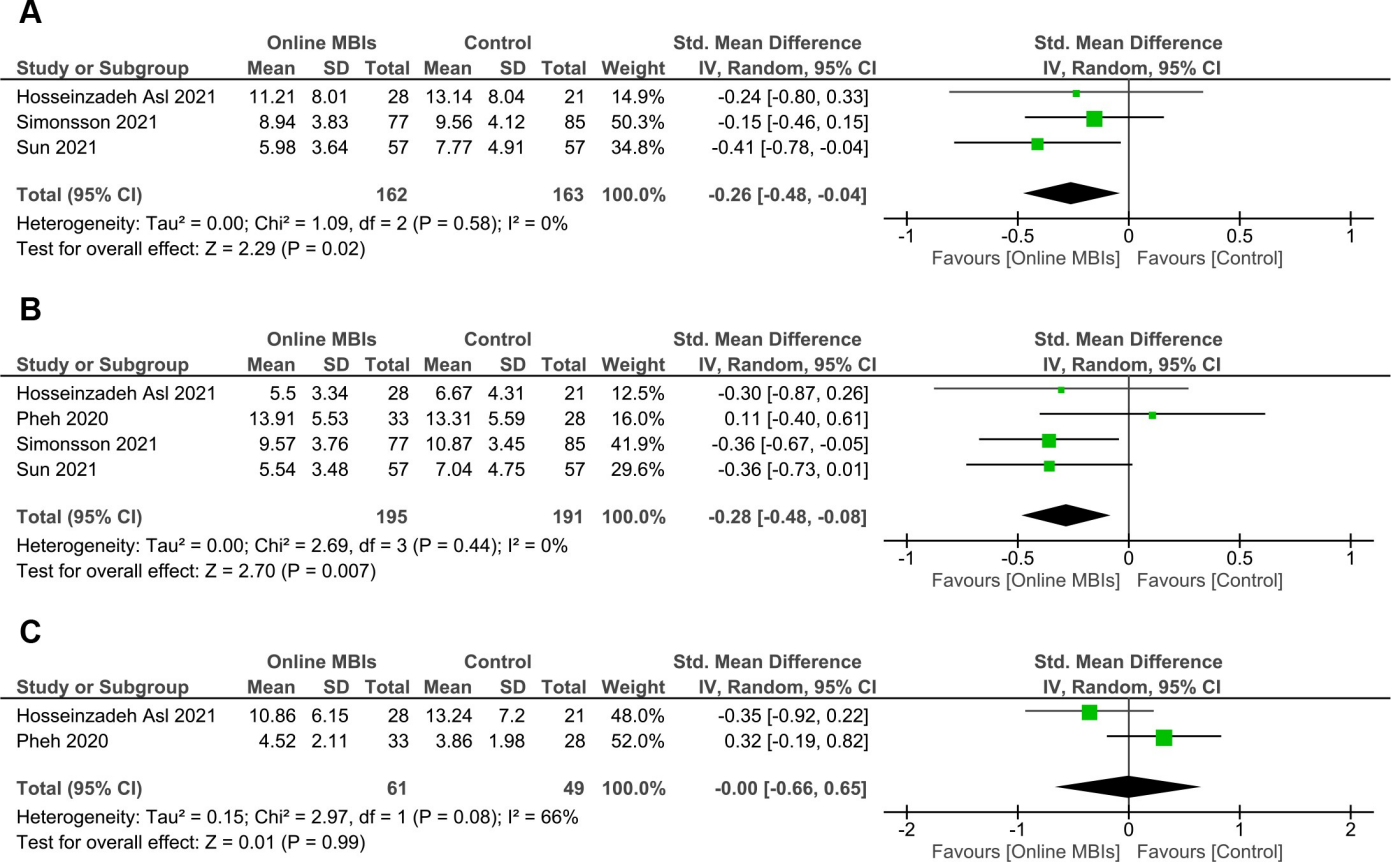
Forest plots of meta-analyses of online MBIs effect on mental health outcomes at follow-up. (**A**) Depression. (**B**) Anxiety. (**C**) Stress.

At follow-up (**Fig 5A**), pooled effect of online MBIs in reducing depression was maintained with a small significant result (*n* = 3; *g* = -0.26; 95% CI = -0.48 to -0.04; *p* = 0.02). Analysis on anxiety outcome at follow-up (**Fig 5B**) also showed a maintained significant, small reduction effect (*n* = 4; *g* = -0.28; 95% CI = -0.48 to -0.08; *p* = 0.007). The level of heterogeneity for depression and anxiety analyses was negligible (*I*^2^ = 0%). For stress (**Fig 5C**), no significant effect at follow-up was found (*n* = 2; *g* = -0.00; 95% CI = -0.66 to 0.65; *p* = 0.99). The heterogeneity was at a high level (*I*^2^ = 66%). The forest plots revealed no study outliers in all outcomes. No potential publication bias was observed in depression and stress outcomes as indicated by the funnel plots (**S3A and S3C Fig in S1 File**) and the results of the Egger’s tests (depression: *Z* = -0.26, *p* = 0.80; stress: *Z* = -0.86, *p* = 0.39). The funnel plot for anxiety outcome was somewhat asymmetric (**S3B Fig in S1 File**). Nonetheless, the Egger’s test result showed no potential publication bias (*Z* = 1.00; *p* = 0.32).

## Discussion

### Main findings

The results of our meta-analysis showed that online MBIs were effective in reducing depression, anxiety, and stress during the COVID-19 pandemic. We found small to moderate reductions in post-intervention depression, anxiety, and stress. Our results are comparable to the previous MBIs studies for clinical and general healthy populations [40, 41]. We also reported significant effects of online MBIs on depression and anxiety at follow-up, suggesting the medium-term effects of online MBIs in improving mental health problems. These findings may be caused by the fact that mindfulness is associated with acceptance skills, which facilitated further mental health resilience [42]. A previous review article by Guendelman et al. [43] also suggested that there were structural and functional changes in the neurons after 8-week mindfulness-based therapies. Evidence has shown an increase in the functional activity of the prefrontal cortex, cingulate cortex, insula, and hippocampus in either healthy or non-healthy participants after receiving MBIs. In addition, the amygdala showed decreased functional activity and an improvement of its connectivity with the prefrontal cortex, leading to better one’s emotional regulation [44]. These changes may be responsible for the strength resilience in patients after MBIs, making these interventions beneficial during the COVID-19 pandemic.

Our study did not exhibit a significant reduction effect on stress at follow-up. This insignificant finding was possibly due to the small number of studies included in the analysis. Moreover, there was a difference in the length of the intervention between the two included studies [33, 36]. The study by Hosseinzadeh Asl et al. [33] used a 4-week online MBI, while Pheh and his colleagues [36] used an ultra-brief online MBI protocol consisting of only one session. The effectiveness of ultra-brief MBIs is still debatable. A preceding study showed a long-lasting positive effect of ultra-brief MBIs [45], while another showed the opposite [46]. Pheh et al. [36] themselves argued that online ultra-brief mindfulness intervention might not be substantial enough to provide long-term changes. Additionally, as described by Mansell et al. [47], ultra-brief mindfulness practices can only dilute stress. The discovery of new perspectives from longer practices is needed to restore better and develop maintained well-being [47, 48].

Subgroup analysis showed that the type of control conditions used to compare the online MBIs potentially affected the pooled effect size for anxiety outcome. We found that studies with inactive controls tended to have larger effect sizes than studies with active controls. The given active interventions, including mental health education, answering manipulated questions, and social support, have been proven to reduce anxiety [49–53]. Therefore, the observed effect sizes seemed to be lower than using inactive controls.

Subgroup analysis for anxiety outcome showed a greater reduction effect for studies conducted outside of Asia than in Asia. This finding may be caused by the difference in racial ethnicities of the study participants between the subgroups [54]. A prior study showed that racial ethnicity affects the use of mindfulness in a weight loss program [55]. Masuda et al. [56] reported that mindfulness interventions could moderate the association between eating disorder cognition and behavior in the White American group, but not in the Asian American or the Black American groups. However, our finding must be carefully interpreted since it contradicted the result from Kahn et al. [57], which revealed that mindfulness is more relevant to Asian populations for regulating emotion. Future online MBIs studies incorporating inter-ethnic analyses are needed to confirm our finding.

According to the meta-regression analyses, we observed a greater anxiety reduction following a longer duration of mindfulness interventions. This finding was confirmed by the sensitivity analysis result after removing Simonsson et al. [37], where this study had the longest MBI duration (8 weeks) of the 6 RCTs included in the analysis. The longer length of psychological interventions will cultivate positive feelings, behaviors, and cognition, leading to an improvement in well-being and other mental health components [48]. Also, repeated mental health interventions in a longer duration will further improve participants’ life satisfaction [58]. We additionally found that age was associated with the effect of online MBIs on stress. As argued by Geiger et al. [59], the MBI is a more well-suited type of mental health intervention for older adults due to its similar concept to their coping and emotional control mechanisms towards mental health problems. Older people as well tend to demonstrate a better acceptance towards mindfulness-based practices [60]. Hence, online MBIs may serve as a promising strategy to face age-related mental health problems in the COVID-19 pandemic era.

### Feasibility of online MBIs during the COVID-19 pandemic

The practice of social distancing and staying home is needed to reduce the transmission of COVID-19 [61, 62]. Hence, online delivered medical interventions have become an essential strategy during the COVID-19 pandemic while still providing the best care for patients [63, 64]. Prior to the pandemic, online MBIs had been in fact considered as promising mental health treatment practices based on a previous meta-analysis by Spijkerman et al. [10]. Nevertheless, our study was more focused on evaluating the role of online MBIs during the COVID-19 pandemic. When compared to the previous meta-analysis [10], our results showed a similar reduction effect for depression, anxiety, and stress, indicating that the implementation of online MBIs is still relevant during the pandemic.

We found that the adherence rates of participants were varied between 86% and 100%, with a pooled rate of 94%. This number was comparable with the previous study’s finding, which revealed an 85% adherence to the multisession online intervention [65]. Despite the lack of studies that reported adherence in online mental health practices, this intervention method has been shown to be helpful and convenient for some participants [66]. The usage of telehealth sessions has also considerably decreased the frequency of missed appointments and dropouts and thus increased the efficiency of mental health care [67]. Although online intervention may not be suitable for some patients with complex and severe mental illnesses, this method is still helpful in fulfilling the need for psychological therapy in the general population.

Additionally, treatment costs may affect the patients’ decisions to seek mental health treatment [68]. A study reported that approximately 57% of obsessive-compulsive disorders patients were unable to afford their treatment costs [69]. Marques and his colleagues [69] also reported complaints of inconvenience from more than a quarter of patients due to spending much time seeking treatment. Moreover, logistic factors such as transportation and treatment schedules also play a role in the patients’ treatment-seeking actions [70]. Ultimately, online-based therapy will help in overcoming economic and adherence-related barriers because of its feasibility and high acceptability, leading to a more convenient method of intervention amidst the COVID-19 crisis [71, 72].

### Strengths and limitations

To the best of our knowledge, this is the first comprehensive systematic review and meta-analysis that examined the effectiveness of online MBIs on depression, anxiety, and stress during the COVID-19 pandemic. We synthesized evidence from available RCTs, where these study designs are regarded as the most suitable and recommended way to evaluate the efficacy of interventions [73]. Furthermore, the total numbers of samples included in most analyses were relatively sufficient, supported by the wide range of areas covered in Asia, America, and Europe, which may strengthen the generalizability of the study’s conclusion. In addition to the short-term effects, we were able to reveal the medium-term effects of online MBIs. Subgroup and meta-regression analyses were also conducted to search for potential variables that could affect the pooled results. Moreover, we identified an ongoing RCT (NCT04720404) [74] with a topic similar to the current study, suggesting the growing interest in implementing online MBIs during a crisis situation like the COVID-19 outbreak.

Although we have made every effort to provide the best possible quality of the study, we also acknowledged that there are still some limitations. First, there was considerable diversity in between-studies characteristics, such as population, instruments used to measure the outcomes, and notably the protocol and method for delivering the MBIs, including the length of follow-up. Although the random-effects model and the SMD of Hedges’ *g* have been employed to minimize the possible variability-related effects, our results should be interpreted with caution. Second, we were not able to determine the long-term effect of online MBIs, given that the length of follow-up reported at most was two months. Third, a publication bias was observed in the post-intervention effect of online MBIs on stress. Nevertheless, through an adequate analysis, we successfully identified that the bias most likely arose from one study by Alvarado-García et al. [32]. Also, the subsequent removal of this study showed no substantial effect on the significance of the pooled result. Fourth, given the small number of studies included in each subgroup, it was not possible to conduct analyses for several covariates. Lastly, the quality of the included studies was yet rather low.

## Conclusion and recommendations

The online MBIs showed promising effects in improving mental health problems, notably depression, anxiety, and stress, during a restricted situation like the COVID-19 pandemic. Although the results were small to moderate, the low cost and ease of access of online MBIs supported with adequate adherence may facilitate them as feasible alternatives to promote mental health in a wide range of communities. In addition, the online MBIs provided maintained improvement on depression and anxiety after follow-up, suggesting their middle-term utility.

Given the growing interest and potential utility of online MBIs in clinical practice, particularly during the COVID-19 pandemic, we encourage more research to investigate further evidence regarding the maximum benefits of online MBIs and the potential influencing factors on the treatment effectiveness with several additional considerations. Future explorations and researchers could look into: (1) determining whether the observed beneficial effects are sustained after longer follow-up periods; (2) evaluating the effects of online MBIs in different patients’ characteristics; (3) identifying subgroups of several factors which could not be analyzed in the current study; and (4) considering biases and quality criteria when conducting studies and publishing results, since the quality of the current included studies were yet unsatisfactory. Finally, we suggest that online MBIs may be incorporated in other forms of online psychotherapies to enhance their utility in improving various mental health issues.

## Supporting information

S1 File Supplementary materials. (PDF)

S2 File PRISMA 2020 checklist. (PDF)

## References

[1] Kaligis F, Indraswari MT, Ismail RI. Stress during COVID-19 pandemic: Mental health condition in Indonesia. Med J Indones. 2020;29: 436–441. doi: 10.13181/mji.bc.204640

[2] Santomauro DF, Mantilla Herrera AM, Shadid J, Zheng P, Ashbaugh C, Pigott DM, et al. Global prevalence and burden of depressive and anxiety disorders in 204 countries and territories in 2020 due to the COVID-19 pandemic. Lancet. 2021;398: 1700–1712. doi: 10.1016/S0140-6736(21)02143-7 34634250 PMC8500697

[3] Chakraborty P, Mittal P, Gupta MS, Yadav S, Arora A. Opinion of students on online education during the COVID-19 pandemic. Hum Behav Emerg Technol. 2021;3: 357–365. doi: 10.1002/hbe2.240

[4] Arora A, Chakraborty P, Bhatia MPS, Mittal P. Role of Emotion in Excessive Use of Twitter During COVID-19 Imposed Lockdown in India. J Technol Behav Sci. 2021;6: 370–377. doi: 10.1007/s41347-020-00174-3 33102690 PMC7572156

[5] Chatterjee I, Chakraborty P. Use of Information Communication Technology by Medical Educators Amid COVID-19 Pandemic and Beyond. J Educ Technol Syst. 2021;49: 310–324. doi: 10.1177/0047239520966996

[6] Arora A, Chakraborty P, Bhatia MPS. Problematic Use of Digital Technologies and Its Impact on Mental Health During COVID-19 Pandemic: Assessment Using Machine Learning BT—Emerging Technologies During the Era of COVID-19 Pandemic. In: Arpaci I, Al-Emran M, A. Al-Sharafi M, Marques G, editors. Emerging Technologies During the Era of COVID-19 Pandemic. Cham: Springer International Publishing; 2021. pp. 197–221. doi: 10.1007/978-3-030-67716-9_13

[7] Liu X, Zhu M, Zhang R, Zhang J, Zhang C, Liu P, et al. Public mental health problems during COVID-19 pandemic: a large-scale meta-analysis of the evidence. Transl Psychiatry. 2021;11: 1–10. doi: 10.1038/s41398-021-01501-9 34244469 PMC8266633

[8] Salari N, Hosseinian-Far A, Jalali R, Vaisi-Raygani A, Rasoulpoor S, Mohammadi M, et al. Prevalence of stress, anxiety, depression among the general population during the COVID-19 pandemic: A systematic review and meta-analysis. Global Health. 2020;16: 1–11. doi: 10.1186/s12992-020-00589-w 32631403 PMC7338126

[9] Zhang D, Lee EKP, Mak ECW, Ho CY, Wong SYS. Mindfulness-based interventions: An overall review. Br Med Bull. 2021;138: 41–57. doi: 10.1093/bmb/ldab005 33884400 PMC8083197

[10] Spijkerman MPJ, Pots WTM, Bohlmeijer ET. Effectiveness of online mindfulness-based interventions in improving mental health: A review and meta-analysis of randomised controlled trials. Clin Psychol Rev. 2016;45: 102–114. doi: 10.1016/j.cpr.2016.03.009 27111302

[11] Shapero BG, Greenberg J, Pedrelli P, de Jong M, Desbordes G. Mindfulness-Based Interventions in Psychiatry. Focus (Madison). 2018;16: 32–39. doi: 10.1176/appi.focus.20170039 29599651 PMC5870875

[12] Goldberg SB, Riordan KM, Sun S, Davidson RJ. The Empirical Status of Mindfulness-Based Interventions: A Systematic Review of 44 Meta-Analyses of Randomized Controlled Trials. Perspect Psychol Sci. 2021. doi: 10.1177/1745691620968771 33593124 PMC8364929

[13] Garfin DR, Cipres AL, Reyes RM. Mindfulness-based interventions to address psychological distress during COVID-19: applications and opportunities. Int J Complement Altern Med. 2021;14: 64–67. doi: 10.15406/ijcam.2021.14.00534

[14] Andersson G, Titov N. Advantages and limitations of Internet-based interventions for common mental disorders. World Psychiatry. 2014;13: 4–11. doi: 10.1002/wps.20083 24497236 PMC3918007

[15] Zhang Y, Xue J, Huang Y. A meta-analysis: Internet mindfulness-based interventions for stress management in the general population. Medicine (Baltimore). 2020;99: e20493. doi: 10.1097/MD.0000000000020493 32664060 PMC7360300

[16] Page MJ, McKenzie JE, Bossuyt PM, Boutron I, Hoffmann TC, Mulrow CD, et al. The PRISMA 2020 statement: An updated guideline for reporting systematic reviews. BMJ. 2021;372. doi: 10.1136/bmj.n71 33782057 PMC8005924

[17] Witarto BS, Visuddho V, Witarto AP. Effectiveness of online mindfulness-based interventions on mental health during the COVID-19 pandemic: a systematic review and meta-analysis of randomized controlled trials. PROSPERO. 2021. Available: https://www.crd.york.ac.uk/prospero/display_record.php?ID=CRD42021287616 10.1371/journal.pone.0274177 PMC9491555 36129900

[18] Adams J, Hillier-Brown FC, Moore HJ, Lake AA, Araujo-Soares V, White M, et al. Searching and synthesising “grey literature” and “grey information” in public health: critical reflections on three case studies. Syst Rev. 2016;5: 164. doi: 10.1186/s13643-016-0337-y 27686611 PMC5041336

[19] Paez A. Grey literature: An important resource in systematic reviews. J Evid Based Med. 2017. doi: 10.1111/jebm.12265 29266844

[20] Watson C. Rise of the preprint: how rapid data sharing during COVID-19 has changed science forever. Nature medicine. United States; 2022. pp. 2–5. doi: 10.1038/s41591-021-01654-6 35031791

[21] Methley AM, Campbell S, Chew-Graham C, McNally R, Cheraghi-Sohi S. PICO, PICOS and SPIDER: A comparison study of specificity and sensitivity in three search tools for qualitative systematic reviews. BMC Health Serv Res. 2014;14. doi: 10.1186/s12913-014-0579-0 25413154 PMC4310146

[22] Cucinotta D, Vanelli M. WHO declares COVID-19 a pandemic. Acta Biomed. 2020;91: 157–160. doi: 10.23750/abm.v91i1.9397 32191675 PMC7569573

[23] Sterne JAC, Savović J, Page MJ, Elbers RG, Blencowe NS, Boutron I, et al. RoB 2: A revised tool for assessing risk of bias in randomised trials. BMJ. 2019;366: 1–8. doi: 10.1136/bmj.l4898 31462531

[24] Nyaga VN, Arbyn M, Aerts M. Metaprop: A Stata command to perform meta-analysis of binomial data. Arch Public Heal. 2014;72: 1–10. doi: 10.1186/2049-3258-72-39 25810908 PMC4373114

[25] Cuijpers P. Meta-analysis in mental health: a practical guide. Pim Cuijpers; 2016.

[26] Higgins J, Thomas J, Chandler J, Cumpston M, T L, Page M, et al. Chapter 10: Analysing data and undertaking meta-analyses. In: Deeks J, Higgins J, Altman D, editors. Cochrane Handbook for Systematic Reviews of Interventions (updated February 2021). Cochrane; 2021.

[27] Higgins J, Thomas J, Chandler J, Cumpston M, T L, Page M, et al. Chapter 6: Choosing effect measures and computing estimates of effect. In: Higgins J, Li T, Deeks J, editors. Cochrane Handbook for Systematic Reviews of Interventions (updated February 2021). Cochrane; 2021.

[28] Cohen J. Statistical Power Analysis for the Behavioral Sciences. 2nd ed. New York: Routledge; 1988.

[29] Higgins JPT, Thompson SG, Deeks JJ, Altman DG. Measuring inconsistency in meta-analyses. BMJ. 2003;327: 557–560. doi: 10.1136/bmj.327.7414.557 12958120 PMC192859

[30] Egger M, Smith GD, Schneider M, Minder C. Bias in meta-analysis detected by a simple, graphical test. Br Med J. 1997;315: 629–634. doi: 10.1136/bmj.315.7109.629 9310563 PMC2127453

[31] Schwarzer G, Carpenter JR, Rücker G. Small-Study Effects in Meta-Analysis BT—Meta-Analysis with R. In: Schwarzer G, Carpenter JR, Rücker G, editors. Cham: Springer International Publishing; 2015. pp. 107–141. doi: 10.1007/978-3-319-21416-0_5

[32] Alvarado-García PAA, Soto-Vásquez MR. Effect of an online mindfulness program on stress during the covid-19 pandemic. Med Natur. 2021;15: 46–49.

[33] Hosseinzadeh Asl NR. A randomized controlled trial of a mindfulness-based intervention in social workers working during the COVID-19 crisis. Curr Psychol. 2021; 1–8. doi: 10.1007/s12144-021-02150-3 34393464 PMC8352152

[34] Huang X, Li J, Yu H, Huang L, Huang X, Wang X. Comparison of Psychological Intervention Effects of Mindfulness-Based Stress Reduction and Mental Health Education on Medical Staff in COVID-19 Isolation Ward. J Chengdu Med Coll. 2021;16: 197–202. doi: 10.3939/j.issn.1674-2257.2021.02.016

[35] Kam JWY, Javed J, Hart CM, Andrews-Hanna JR, Tomfohr-Madsen LM, Mills C. Daily mindfulness training reduces negative impact of COVID-19 news exposure on affective well-being. Psychol Res. 2021; 1–12. doi: 10.1007/s00426-021-01550-1 34165612 PMC8222951

[36] Pheh K-S, Tan H-C, Tan C-S. Effects of an Ultra-brief Online Mindfulness-based Intervention on Mental Health during the Coronavirus Disease (COVID-19) Outbreak in Malaysia: A Randomized Controlled Trial. Makara Hum Behav Stud Asia. 2020;24: 118. doi: 10.7454/hubs.asia.2140920

[37] Simonsson O, Bazin O, Fisher SD, Goldberg SB. Effects of an eight-week, online mindfulness program on anxiety and depression in university students during COVID-19: A randomized controlled trial. Psychiatry Res. 2021;305: 114222. doi: 10.1016/j.psychres.2021.114222 34601450 PMC8459547

[38] Smith RB, Mahnert ND, Foote J, Saunders KT, Mourad J, Huberty J. Mindfulness Effects in Obstetric and Gynecology Patients During the Coronavirus Disease 2019 (COVID-19) Pandemic: A Randomized Controlled Trial. Obstet Gynecol. 2021;137: 1032–1040. doi: 10.1097/AOG.0000000000004316 33957663 PMC8132566

[39] Sun S, Lin D, Goldberg S, Shen Z, Chen P, Qiao S, et al. A mindfulness-based mobile health (mHealth) intervention among psychologically distressed university students in quarantine during the COVID-19 pandemic: A randomized controlled trial. J Couns Psychol. 2021. doi: 10.1037/cou0000568 34264696 PMC8760365

[40] Khoury B, Sharma M, Rush SE, Fournier C. Mindfulness-based stress reduction for healthy individuals: A meta-analysis. J Psychosom Res. 2015;78: 519–528. doi: 10.1016/j.jpsychores.2015.03.009 25818837

[41] Huberty J, Green J, Glissmann C, Larkey L, Puzia M, Lee C. Efficacy of the Mindfulness Meditation Mobile App “Calm” to Reduce Stress Among College Students: Randomized Controlled Trial. JMIR mHealth uHealth. 2019;7: e14273. doi: 10.2196/14273 31237569 PMC6614998

[42] Chin B, Lindsay EK, Greco CM, Brown KW, Smyth JM, Wright AGC, et al. Psychological mechanisms driving stress resilience in mindfulness training: A randomized controlled trial. Heal Psychol Off J Div Heal Psychol Am Psychol Assoc. 2019;38: 759–768. doi: 10.1037/hea0000763 31120272 PMC6681655

[43] Guendelman S, Medeiros S, Rampes H. Mindfulness and Emotion Regulation: Insights from Neurobiological, Psychological, and Clinical Studies. Front Psychol. 2017;8: 220. doi: 10.3389/fpsyg.2017.00220 28321194 PMC5337506

[44] Gotink RA, Meijboom R, Vernooij MW, Smits M, Hunink MGM. 8-week Mindfulness Based Stress Reduction induces brain changes similar to traditional long-term meditation practice—A systematic review. Brain Cogn. 2016;108: 32–41. doi: 10.1016/j.bandc.2016.07.001 27429096

[45] Shuai R, Bakou AE, Hardy L, Hogarth L. Ultra-brief breath counting (mindfulness) training promotes recovery from stress-induced alcohol-seeking in student drinkers. Addict Behav. 2020;102: 106141. doi: 10.1016/j.addbeh.2019.106141 31704429 PMC6959458

[46] Elefant AB, Contreras O, Muñoz RF, Bunge EL, Leykin Y. Microinterventions produce immediate but not lasting benefits in mood and distress. Internet Interv. 2017;10: 17–22. doi: 10.1016/j.invent.2017.08.004 29270366 PMC5734669

[47] Mansell W, Urmson R, Mansell L. The 4Ds of Dealing With Distress—Distract, Dilute, Develop, and Discover: An Ultra-Brief Intervention for Occupational and Academic Stress. Front Psychol. 2020;11: 611156. doi: 10.3389/fpsyg.2020.611156 33391129 PMC7772151

[48] Sin NL, Lyubomirsky S. Enhancing well-being and alleviating depressive symptoms with positive psychology interventions: a practice-friendly meta-analysis. J Clin Psychol. 2009;65: 467–487. doi: 10.1002/jclp.20593 19301241

[49] Lattie EG, Adkins EC, Winquist N, Stiles-Shields C, Wafford QE, Graham AK. Digital Mental Health Interventions for Depression, Anxiety, and Enhancement of Psychological Well-Being Among College Students: Systematic Review. J Med Internet Res. 2019;21: e12869. doi: 10.2196/12869 31333198 PMC6681642

[50] Deolmi M, Pisani F. Psychological and psychiatric impact of COVID-19 pandemic among children and adolescents. Acta Biomed. 2020;91: e2020149. doi: 10.23750/abm.v91i4.10870 33525229 PMC7927507

[51] Shao R, He P, Ling B, Tan L, Xu L, Hou Y, et al. Prevalence of depression and anxiety and correlations between depression, anxiety, family functioning, social support and coping styles among Chinese medical students. BMC Psychol. 2020;8: 38. doi: 10.1186/s40359-020-00402-8 32321593 PMC7178943

[52] Labrague LJ, De Los Santos JAA. COVID-19 anxiety among front-line nurses: Predictive role of organisational support, personal resilience and social support. J Nurs Manag. 2020;28: 1653–1661. doi: 10.1111/jonm.13121 32770780 PMC7436313

[53] Chen S-P, Chang W-P, Stuart H. Self-reflection and screening mental health on Canadian campuses: validation of the mental health continuum model. BMC Psychol. 2020;8: 76. doi: 10.1186/s40359-020-00446-w 32727614 PMC7391623

[54] Waldron EM, Hong S, Moskowitz JT, Burnett-Zeigler I. A Systematic Review of the Demographic Characteristics of Participants in US-Based Randomized Controlled Trials of Mindfulness-Based Interventions. Mindfulness (N Y). 2018;9: 1671–1692. doi: 10.1007/s12671-018-0920-5

[55] Daubenmier J, Chao MT, Hartogensis W, Liu R, Moran PJ, Acree MC, et al. Exploratory Analysis of Racial/Ethnic and Educational Differences in a Randomized Controlled Trial of a Mindfulness-Based Weight Loss Intervention. Psychosom Med. 2021;83. 10.1097/PSY.0000000000000859 33214537

[56] Masuda A, Price M, Latzman RD. Mindfulness Moderates the Relationship Between Disordered Eating Cognitions and Disordered Eating Behaviors in a Non-Clinical College Sample. J Psychopathol Behav Assess. 2012;34: 107–115. doi: 10.1007/s10862-011-9252-7 22888181 PMC3415312

[57] Kahn JH, Wei M, Su JC, Han S, Strojewska A. Distress disclosure and psychological functioning among taiwanese nationals and european americans: The moderating roles of mindfulness and nationality. J Couns Psychol. 2017;64: 292–301. doi: 10.1037/cou0000202 28240918

[58] Lo ETT, Leung F. Cultivating a Gratitude Thinking Habit and Exploring Its Effects on Psychological Well-being: An Exploratory Longitudinal Study. PsyArXiv. 2020. doi: 10.31234/osf.io/hkwsr

[59] Geiger PJ, Boggero IA, Brake CA, Caldera CA, Combs HL, Peters JR, et al. Mindfulness-Based Interventions for Older Adults: A Review of the Effects on Physical and Emotional Well-being. Mindfulness (N Y). 2016;7: 296–307. doi: 10.1007/s12671-015-0444-1 27200109 PMC4868399

[60] Karlin BE, Walser RD, Yesavage J, Zhang A, Trockel M, Taylor CB. Effectiveness of acceptance and commitment therapy for depression: comparison among older and younger veterans. Aging Ment Health. 2013;17: 555–563. doi: 10.1080/13607863.2013.789002 23607328

[61] Chu DK, Akl EA, Duda S, Solo K, Yaacoub S, Schünemann HJ. Physical distancing, face masks, and eye protection to prevent person-to-person transmission of SARS-CoV-2 and COVID-19: a systematic review and meta-analysis. Lancet (London, England). 2020;395: 1973–1987. doi: 10.1016/S0140-6736(20)31142-9 32497510 PMC7263814

[62] Kotwal AA, Holt-Lunstad J, Newmark RL, Cenzer I, Smith AK, Covinsky KE, et al. Social Isolation and Loneliness Among San Francisco Bay Area Older Adults During the COVID-19 Shelter-in-Place Orders. J Am Geriatr Soc. 2021;69: 20–29. doi: 10.1111/jgs.16865 32965024 PMC7536935

[63] MahajanV, SinghT, AzadC. Using Telemedicine During the COVID-19 Pandemic. Indian Pediatr. 2020;57: 652–657. 32412914

[64] Weiner L, Berna F, Nourry N, Severac F, Vidailhet P, Mengin AC. Efficacy of an online cognitive behavioral therapy program developed for healthcare workers during the COVID-19 pandemic: the REduction of STress (REST) study protocol for a randomized controlled trial. Trials. 2020;21: 870. doi: 10.1186/s13063-020-04772-7 33087178 PMC7576984

[65] Beintner I, Vollert B, Zarski A-C, Bolinski F, Musiat P, Görlich D, et al. Adherence Reporting in Randomized Controlled Trials Examining Manualized Multisession Online Interventions: Systematic Review of Practices and Proposal for Reporting Standards. J Med Internet Res. 2019;21: e14181. doi: 10.2196/14181 31414664 PMC6713038

[66] Achilles MR, Anderson M, Li SH, Subotic-Kerry M, Parker B, O’Dea B. Adherence to e-mental health among youth: Considerations for intervention development and research design. Digit Heal. 2020;6: 2055207620926064. doi: 10.1177/2055207620926064 32547775 PMC7249594

[67] Reay RE, Looi JC, Keightley P. Telehealth mental health services during COVID-19: summary of evidence and clinical practice. Australas psychiatry Bull R Aust New Zeal Coll Psychiatr. 2020;28: 514–516. doi: 10.1177/1039856220943032 32722963 PMC7387833

[68] Roberts T, Miguel Esponda G, Krupchanka D, Shidhaye R, Patel V, Rathod S. Factors associated with health service utilisation for common mental disorders: A systematic review. BMC Psychiatry. 2018;18: 1–19. doi: 10.1186/s12888-018-1837-1 30134869 PMC6104009

[69] Marques L, LeBlanc NJ, Weingarden HM, Timpano KR, Jenike M, Wilhelm S. Barriers to treatment and service utilization in an internet sample of individuals with obsessive-compulsive symptoms. Depress Anxiety. 2010;27: 470–475. doi: 10.1002/da.20694 20455248

[70] Radez J, Reardon T, Creswell C, Lawrence PJ, Evdoka-Burton G, Waite P. Why do children and adolescents (not) seek and access professional help for their mental health problems? A systematic review of quantitative and qualitative studies. Eur Child Adolesc Psychiatry. 2021;30: 183–211. doi: 10.1007/s00787-019-01469-4 31965309 PMC7932953

[71] Schleider JL, Dobias M, Sung J, Mumper E, Mullarkey MC. Acceptability and Utility of an Open-Access, Online Single-Session Intervention Platform for Adolescent Mental Health. JMIR Ment Heal. 2020;7: e20513. doi: 10.2196/20513 32602846 PMC7367540

[72] Scholl J, Kohls E, Görges F, Steinbrecher M, Baldofski S, Moessner M, et al. Acceptability and Feasibility of the Transfer of Face-to-Face Group Therapy to Online Group Chats in a Psychiatric Outpatient Setting During the COVID-19 Pandemic: Longitudinal Observational Study. JMIR Form Res. 2021;5: e27865. doi: 10.2196/27865 34161252 PMC8315157

[73] Kabisch M, Ruckes C, Seibert-Grafe M, Blettner M. Randomized controlled trials: part 17 of a series on evaluation of scientific publications. Dtsch Arztebl Int. 2011;108: 663–668. doi: 10.3238/arztebl.2011.0663 22013494 PMC3196997

[74] Speckens A. Mindful Prevention of Psychopathology in Healthcare Workers During the COVID-19 Crisis (COVID-19 MindPreP). ClinicalTrials.gov. 2021. Available: https://clinicaltrials.gov/ct2/show/NCT04720404

