# Supplementary material for: Effectiveness of online mindfulness-based interventions in improving mental health during the COVID-19 pandemic: A systematic review and meta-analysis of randomized controlled trials
Source: PLoS One. 2022 Sep 21;17(9):e0274177. doi: 10.1371/journal.pone.0274177 (PMC9491555; doi:10.1371/journal.pone.0274177)
Supplement: S1 File — (PDF) [file pone.0274177.s001.pdf]

SUPPLEMENTARY MATERIALS

Effectiveness of online mindfulness-based interventions in improving mental health during the COVID-19 pandemic: A systematic review and meta-analysis of randomized controlled trials

Bendix Samarta Witarto[1], Visuddho Visuddho[1], Andro Pramana Witarto[1], Damba Bestari[2,3], Brihastami Sawitri[2,3], Tando Abner Sivile Melapi[4], Citrawati Dyah Kencono Wungu[5,6*]

[1] Medical Program, Faculty of Medicine, Universitas Airlangga, Surabaya, Indonesia
[2] Department of Psychiatry, Faculty of Medicine Universitas Airlangga/Dr. Soetomo Hospital, Surabaya, Indonesia
[3] Department of Psychiatry, Universitas Airlangga Hospital, Surabaya, Indonesia
[4] Department of Psychiatry, University of the Witwatersrand, Johannesburg, South Africa
[5] Department of Physiology and Medical Biochemistry, Faculty of Medicine, Universitas Airlangga, Surabaya, Indonesia
[6] Institute of Tropical Disease, Universitas Airlangga, Surabaya, Indonesia

*** Corresponding author**
Citrawati Dyah Kencono Wungu, Department of Physiology and Medical Biochemistry, Faculty of Medicine, Universitas Airlangga, Jl. Mayjen Prof. Dr. Moestopo 47, Surabaya, East Java 60132, Indonesia. Email: citrawati.dyah@fk.unair.ac.id

TABLE OF CONTENTS

Supplementary Material	Page
**Title, Authors, and Affiliations Page**	1
**Table of Contents**	2
**S1 Table.** Search strategies.	3
**S2 Table.** PICOS framework.	5
**S3 Table.** Outcomes of included studies.	6
**S1 Fig.** Bubble plots of meta-regression analyses of online MBIs effect on mental health outcomes at post-intervention.	7
**S2 Fig.** Funnel plots of meta-analyses of online MBIs effect on mental health outcomes at post-intervention.	8
**S3 Fig.** Funnel plots of meta-analyses of online MBIs effect on mental health outcomes at follow-up.	9
**References**	10

S1 Table. Search strategies.

Database		Keywords
**PubMed**	#1	"Mindfulness"[MeSH Terms] OR "mindful*"[All Fields]
	#2	"Online Systems"[MeSH Terms] OR "online"[All Fields] OR "on-line"[All Fields] OR "ehealth"[All Fields] OR "e-health"[All Fields] OR "virtual*"[All Fields] OR "web"[All Fields] OR "webs"[All Fields] OR "website*"[All Fields] OR "Internet"[MeSH Terms] OR "internet*"[All Fields] OR "app"[All Fields] OR "apps"[All Fields] OR "application*"[All Fields] OR "mobile*"[All Fields] OR "mhealth"[All Fields] OR "m-health"[All Fields]
	#3	"COVID-19"[MeSH Terms] OR "COVID 19"[All Fields] OR "SARS-COV-2"[MeSH Terms] OR "SARS-COV-2"[All Fields] OR "SARSCoV2"[All Fields] OR "2019-nCoV"[All Fields] OR "2019nCoV"[All Fields] OR "nCoV-2019"[All Fields] OR "nCoV2019"[All Fields] OR "coronavirus disease 2019"[All Fields] OR "novel coronavirus"[All Fields] OR "new coronavirus"[All Fields]
	#4	controlled clinical trial[MeSH Terms] OR randomized controlled trial[MeSH Terms] OR "random allocation"[MeSH Terms] OR "RCT"[All Fields] OR "random*"[All Fields] OR "control*"[All Fields] OR "trial*"[All Fields]
	#5	#1 AND #2 AND #3 AND #4
**Scopus**	#1	TITLE-ABS-KEY("mindful*")
	#2	TITLE-ABS-KEY("online" OR "on-line" OR "ehealth" OR "e-health" OR "virtual*" OR "web*" OR "internet*" OR "app*" OR "mobile*" OR "mhealth" OR "m-health")
	#3	TITLE-ABS-KEY("COVID-19" OR "COVID19" OR "SARS-CoV-2" OR "SARSCoV2" OR "SARS-Cov-19" OR "SARSCoV19" OR "2019-nCoV" OR "2019nCoV" OR "nCoV-2019" OR "nCoV2019" OR "coronavirus disease 2019" OR "novel coronavirus" OR "new coronavirus")
	#4	TITLE-ABS-KEY("RCT" OR "random*" OR "control*" OR "trial*")
	#5	#1 AND #2 AND #3 AND #4
**Web of Science**	#1	ALL=("mindful*")
	#2	ALL=("online" OR "on-line" OR "ehealth" OR "e-health" OR "virtual*" OR "web*" OR "internet*" OR "app*" OR "mobile*" OR "mhealth" OR "m-health")
	#3	ALL=("COVID-19" OR "COVID19" OR "SARS-CoV-2" OR "SARSCoV2" OR "SARS-Cov-19" OR "SARSCoV19" OR "2019-nCoV" OR "2019nCoV" OR "nCoV-2019" OR "nCoV2019" OR "coronavirus disease 2019" OR "novel coronavirus" OR "new coronavirus")
	#4	ALL=("RCT" OR "random*" OR "control*" OR "trial*")
	#5	#1 AND #2 AND #3 AND #4
**ProQuest**	#1	mesh.Exact("mindfulness")
	#2	noft("mindful*")
	#3	mesh.Exact("online systems" OR "internet")
	#4	noft("online" OR "on-line" OR "ehealth" OR "e-health" OR "virtual*" OR "web*" OR "internet*" OR "app*" OR "mobile*" OR "mhealth" OR "m-health")
	#5	noft("COVID-19" OR "COVID19" OR "SARS-CoV-2" OR "SARSCoV2" OR "SARS-Cov-19" OR "SARSCoV19" OR "2019-nCoV" OR "2019nCoV" OR "nCoV-2019" OR "nCoV2019" OR "coronavirus disease 2019" OR "novel coronavirus" OR "new coronavirus")
	#6	mesh.Exact("Controlled Clinical Trial as Topic" OR "Randomized Controlled Trial as Topic" OR "Random Allocation")
	#7	noft("RCT" OR "random*" OR "control*" OR "trial*")
	#8	(#1 OR #2) AND (#3 OR #4) AND #5 AND (#6 OR #7)
**CINAHL (via EBSCOhost)**	#1	(MM "Mindfulness") OR "mindful*"
	#2	(MH "Online Systems+") OR (MH "Internet+") OR (MM "Internet-Based Intervention") OR "online" OR "on-line" OR "ehealth" OR "e-health" OR "virtual*" OR "web" OR "webs" OR "website*" OR "internet*" OR "app" OR "apps" OR "application*" OR "mobile*" OR "mhealth" OR "m-health"
	#3	(MM "COVID-19") OR (MM "SARS-CoV-2") OR "COVID-19" OR "COVID19" OR "SARS-CoV-2" OR "SARSCoV2" OR "SARS-Cov-19" OR "SARSCoV19" OR "2019-nCoV" OR "2019nCoV" OR "nCoV-2019" OR "nCoV2019" OR "coronavirus disease 2019" OR "novel coronavirus" OR "new coronavirus"
	#4	(MH "Clinical Trials+") OR (MH "Randomized Controlled Trials+") OR "RCT" OR "random*" OR "control*" OR "trial*"
	#5	#1 AND #2 AND #3 AND #4
**CENTRAL**	#1	MeSH descriptor: [Mindfulness] explode all trees
	#2	"mindful*"
	#3	MeSH descriptor: [Online Systems] explode all trees
	#4	MeSH descriptor: [Internet] explode all trees
	#5	"online" OR "on-line" OR "ehealth" OR "e-health" OR "virtual*" OR "web*" OR "internet*" OR "app*" OR "mobile*" OR "mhealth" OR "m-health"
	#6	MeSH descriptor: [COVID-19] explode all trees
	#7	MeSH descriptor: [SARS-COV-2] explode all trees

	#8	"COVID-19" OR "COVID19" OR "SARS-CoV-2" OR "SARSCoV2" OR "SARS-Cov-19" OR "SARSCoV19" OR "2019-nCoV" OR "2019nCoV" OR "nCoV-2019" OR "nCoV2019" OR "coronavirus disease 2019" OR "novel coronavirus" OR "new coronavirus"
	#9	MeSH descriptor: [Controlled Clinical Trial] explode all trees
	#10	MeSH descriptor: [Randomized Controlled Trial] explode all trees
	#11	MeSH descriptor: [Random Allocation] explode all trees
	#12	"RCT" OR "random*" OR "control*" OR "trial*"
	#13	#1 OR #2
	#14	#3 OR #4 OR #5
	#15	#6 OR #7 OR #8
	#16	#9 OR #10 OR #11 OR #12
	#17	#13 AND #14 AND #15 AND #16
**WHO COVID-19 Research Database**	#1	tw:("mindful" OR "mindfulness")
	#2	tw:("online" OR "on-line" OR "ehealth" OR "e-health" OR "virtual*" OR "web" OR "webs" OR "website" OR "websites" OR "internet" OR "app" OR "apps" OR "application" OR "applications" OR "mobile" OR "mhealth" OR "m-health")
	#3	tw:("COVID-19" OR "COVID19" OR "SARS-CoV-2" OR "SARSCoV2" OR "SARS-Cov-19" OR "SARSCoV19" OR "2019-nCoV" OR "2019nCoV" OR "nCoV-2019" OR "nCoV2019" OR "coronavirus disease 2019" OR "novel coronavirus" OR "new coronavirus")
	#4	tw:("RCT" OR "random" OR "randomized" OR "randomised" OR "control" OR "controls" OR "controlled" OR "trial" OR "trials")
	#5	#1 AND #2 AND #3 AND #4
**MedRxiv and BioRxiv**	("mindfulness") AND ("online" OR "internet" OR "application") AND ("COVID-19") AND ("randomized" OR "trial")	

CENTRAL, Cochrane Central Register of Controlled Trials; **CINAHL**, Cumulative Index to Nursing and Allied Health Literature; **COVID-19**, Coronavirus Disease 2019; **WHO**, World Health Organization.

S2 Table. PICOS framework.

Components of PICOS	Definition
**P**opulation	Adults aged 18 years and older
**I**ntervention	Online MBIs (including MBSR or MBCT)
**C**omparison	Active or inactive control group
**O**utcome	To reduce symptoms of depression, anxiety, or stress
**S**tudy Design	RCT conducted during the period of the COVID-19 pandemic

COVID-19; Coronavirus Disease 2019; **MBI**, mindfulness-based intervention; **MBCT**, mindfulness-based cognitive therapy; **MBSR**; mindfulness-based stress reduction; **PICOS**, Population, Intervention, Comparison, Outcome, and Study Design; **RCT**, randomized controlled trial.

S3 Table. Outcomes of included studies.

First Author, Year	Depression (Online MBIs)				Depression (Control)				Anxiety (Online MBIs)				Anxiety (Control)				Stress (Online MBIs)				Stress (Control)			
	Post-Test		Follow-Up		Post-Test		Follow-Up		Post-Test		Follow-Up		Post-Test		Post-Test		Follow-Up		Post-Test		Follow-Up		Post-Test	
	M	SD	M	SD	M	SD	M	SD	M	SD	M	SD	M	SD	M	SD	M	SD	M	SD	M	SD	M	SD
**Alvarado-García et al., 2021** [1]	-	-	-	-	-	-	-	-	-	-	-	-	-	-	-	-	37.83	6.07	-	-	50.58	8.12	-	-
**Hosseinzadeh Asl, 2021** [2]	9.5	8.03	11.21	8.01	12.1	8.89	13.14	8.04	4.79	4.05	5.5	3.34	6	4.35	6.67	4.31	9.93	6.7	10.86	6.15	12.48	7.59	13.24	7.2
**Huang et al., 2021** [3]	-	-	-	-	-	-	-	-	-	-	-	-	-	-	-	-	50.25	30.12	-	-	59.17	11.24	-	-
**Kam et al., 2021** [4]	15.84	7.04	-	-	16.57	6.89	-	-	17.84	7.66	-	-	19.07	8.33	-	-	-	-	-	-	-	-	-	-
**Pheh et al, 2020** [5]	-	-	-	-	-	-	-	-	12.59	4.94	13.91	5.53	12.92	4.8	13.31	5.59	4.02	1.58	4.52	2.11	4.22	1.86	3.86	1.98
**Simonsson et al., 2021** [6]	8.81	3.75	8.94	3.83	10.23	4.06	9.56	4.12	9.81	3.54	9.57	3.76	11.7	3.72	10.87	3.45	-	-	-	-	-	-	-	-
**Smith et al., 2021** [7]	4	2.9	-	-	5.4	3.4	-	-	5	3.9	-	-	6.9	4.7	-	-	12.7	5.6	-	-	17	6.7	-	-
**Sun et al., 2021** [8]	6.42	3.76	5.98	3.64	7.63	5.24	7.77	4.91	6.08	3.99	5.54	3.48	6.13	4.26	7.04	4.75	-	-	-	-	-	-	-	-

M, mean; **MBI**, mindfulness-based intervention; **SD**, standard deviation.

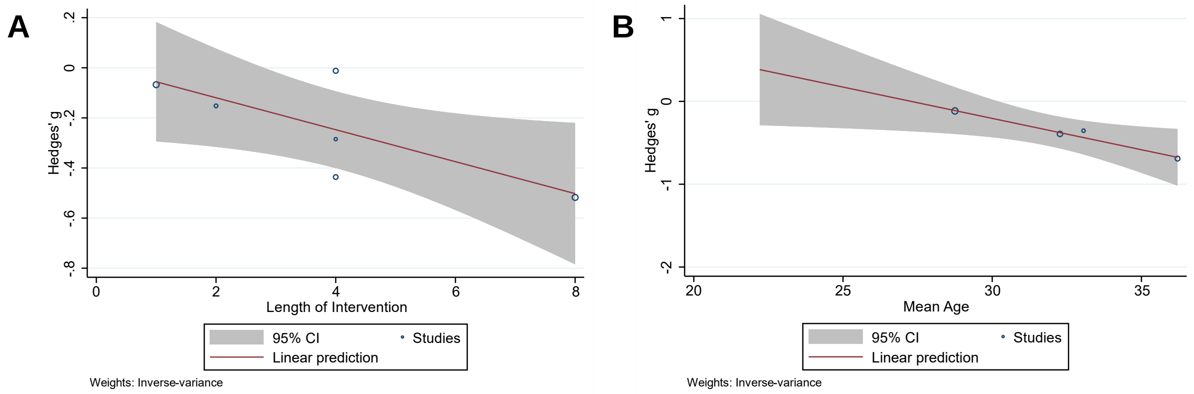

S1 Fig. Bubble plots of meta-regression analyses of online MBIs effect on mental health outcomes at post-intervention. (**A**) Length of intervention on anxiety. (**B**) Mean age on stress.

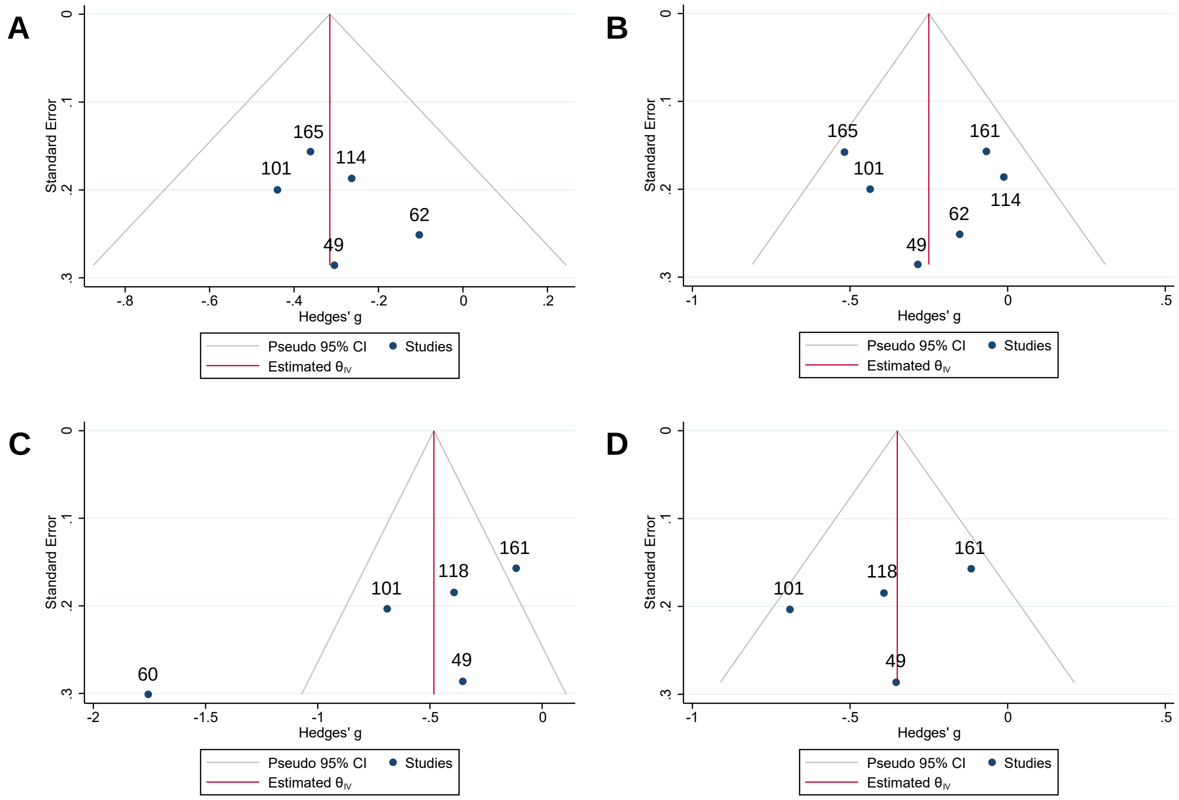

S2 Fig. Funnel plots of meta-analyses of online MBIs effect on mental health outcomes at post-intervention. (**A**) Depression. (**B**) Anxiety. (**C**) Stress (including outliers). (**D**) Stress (excluding outliers). The number above or below each dot indicates the sample size of the corresponding study in the analysis.

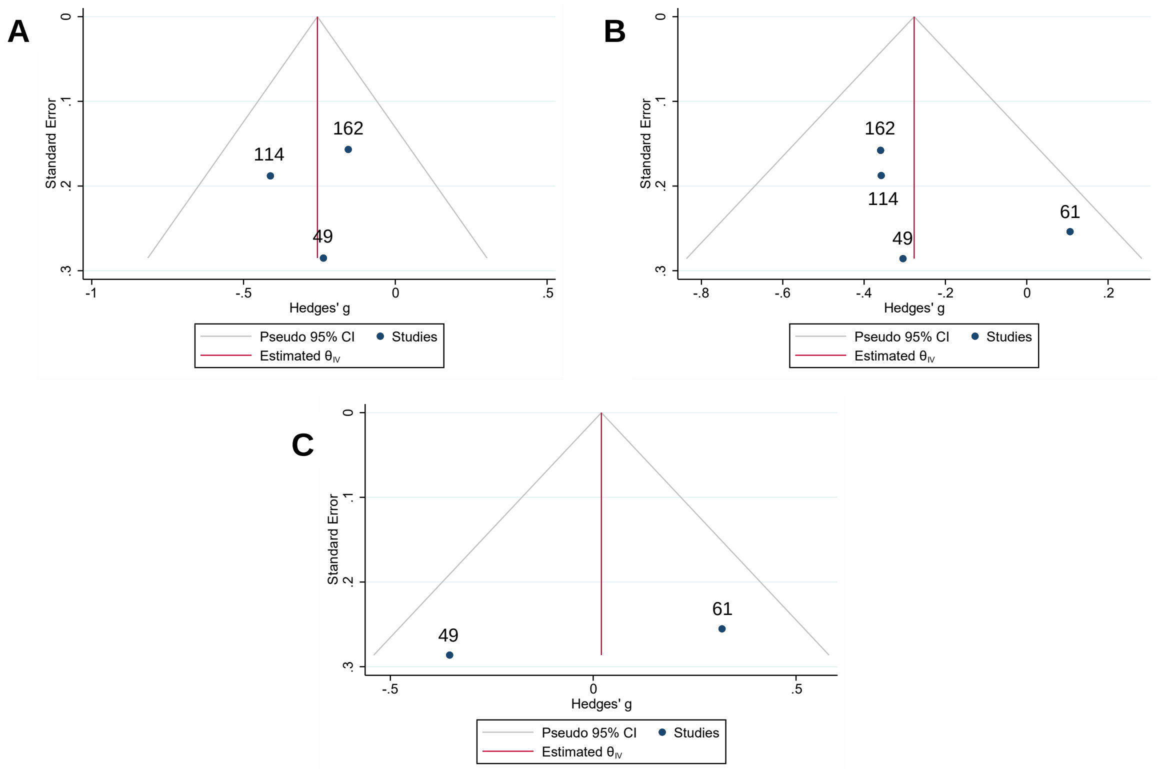

S3 Fig. Funnel plots of meta-analyses of online MBIs effect on mental health outcomes at follow-up. (**A**) Depression. (**B**) Anxiety. (**C**) Stress. The number above or below each dot indicates the sample size of the corresponding study included in the analysis.

REFERENCES

1. Alvarado-García PAA, Soto-Vásquez MR. Effect of an online mindfulness program on stress during the covid-19 pandemic. Med Natur. 2021;15: 46–49.
2. Hosseinzadeh Asl NR. A randomized controlled trial of a mindfulness-based intervention in social workers working during the COVID-19 crisis. Curr Psychol. 2021; 1–8. doi:10.1007/s12144-021-02150-3
3. Huang X, Li J, Yu H, Huang L, Huang X, Wang X. Comparison of Psychological Intervention Effects of Mindfulness-Based Stress Reduction and Mental Health Education on Medical Staff in COVID-19 Isolation Ward. J Chengdu Med Coll. 2021;16: 197–202. doi:10.3939/j.issn.1674-2257.2021.02.016
4. Kam JWY, Javed J, Hart CM, Andrews-Hanna JR, Tomfohr-Madsen LM, Mills C. Daily mindfulness training reduces negative impact of COVID-19 news exposure on affective well-being. Psychol Res. 2021; 1–12. doi:10.1007/s00426-021-01550-1
5. Pheh K-S, Tan H-C, Tan C-S. Effects of an Ultra-brief Online Mindfulness-based Intervention on Mental Health during the Coronavirus Disease (COVID-19) Outbreak in Malaysia: A Randomized Controlled Trial. Makara Hum Behav Stud Asia. 2020;24: 118. doi:10.7454/hubs.asia.2140920
6. Simonsson O, Bazin O, Fisher SD, Goldberg SB. Effects of an eight-week, online mindfulness program on anxiety and depression in university students during COVID-19: A randomized controlled trial. Psychiatry Res. 2021;305: 114222. doi:10.1016/j.psychres.2021.114222
7. Smith RB, Mahnert ND, Foote J, Saunders KT, Mourad J, Huberty J. Mindfulness Effects in Obstetric and Gynecology Patients During the Coronavirus Disease 2019 (COVID-19) Pandemic: A Randomized Controlled Trial. Obstet Gynecol. 2021;137: 1032–1040. doi:10.1097/AOG.0000000000004316
8. Sun S, Lin D, Goldberg S, Shen Z, Chen P, Qiao S, et al. A mindfulness-based mobile health (mHealth) intervention among psychologically distressed university students in quarantine during the COVID-19 pandemic: A randomized controlled trial. J Couns Psychol. 2021. doi:10.1037/cou0000568
